# Dissecting the molecular determinants of clinical PARP1 inhibitor selectivity for tankyrase1

**DOI:** 10.1074/jbc.RA120.016573

**Published:** 2021-01-09

**Authors:** Kevin Ryan, Ben Bolaňos, Marissa Smith, Prakash B. Palde, Paulina Delgado Cuenca, Todd L. VanArsdale, Sherry Niessen, Lianglin Zhang, Douglas Behenna, Martha A. Ornelas, Khanh T. Tran, Stephen Kaiser, Lawrence Lum, Al Stewart, Ketan S. Gajiwala

**Affiliations:** 1 Structural Biology and Protein Science, Pfizer Worldwide Research and Development, San Diego, California, USA; 2 Oncology Research Unit, Pfizer Worldwide Research and Development, San Diego, California, USA; 3 Oncology Medicinal Chemistry, Pfizer Worldwide Research and Development, San Diego, California, USA

**Keywords:** PARP1, tankyrase1, talazoparib, olaparib, niraparib, veliparib, drug design, crystal structure, surface plasmon resonance (SPR), protein–ligand interaction, anticancer drug, hydrogen-deuterium exchange, ARTs, ADP-ribosyltransferases, BRCA1/2, breast cancer susceptibility proteins 1 and 2, D-loop, donor loop, DMSO, dimethyl sulfoxide, HDX–MS, hydrogen–deuterium exchange mass spectrometry, PARP1, poly(ADP-ribosyl) polymerase 1, PDB, Protein Data Bank, SPR, surface plasmon resonance, TCEP, tris(2-carboxyethyl)phosphine, TNKS1, tankyrase1

## Abstract

Poly-ADP-ribosyltransferases play a critical role in DNA repair and cell death, and poly(ADP-ribosyl) polymerase 1 (PARP1) is a particularly important therapeutic target for the treatment of breast cancer because of its synthetic lethal relationship with breast cancer susceptibility proteins 1 and 2. Numerous PARP1 inhibitors have been developed, and their efficacy in cancer treatment is attributed to both the inhibition of enzymatic activity and their ability to trap PARP1 on to the damaged DNA, which is cytotoxic. Of the clinical PARP inhibitors, talazoparib is the most effective at trapping PARP1 on damaged DNA. Biochemically, talazoparib is also suspected to be a potent inhibitor of PARP5a/b (tankyrase1/2 [TNKS1/2]), which is an important regulator of Wnt/β-catenin pathway. Here we show using competition experiments in cell lysate that, at a clinically relevant concentration, talazoparib can potentially bind and engage TNKS1. Using surface plasmon resonance, we measured the dissociation constants of talazoparib, olaparib, niraparib, and veliparib for their interaction with PARP1 and TNKS1. The results show that talazoparib has strong affinity for PARP1 as well as uniquely strong affinity for TNKS1. Finally, we used crystallography and hydrogen deuterium exchange mass spectroscopy to dissect the molecular mechanism of differential selectivity of these PARP1 inhibitors. From these data, we conclude that subtle differences between the ligand-binding sites of PARP1 and TNKS1, differences in the electrostatic nature of the ligands, protein dynamics, and ligand conformational energetics contribute to the different pharmacology of these PARP1 inhibitors. These results will help in the design of drugs to treat Wnt/β-catenin pathway–related cancers, such as colorectal cancers.

ADP-ribosyltransferases (ARTs) are a family of 17 enzymes with a structurally conserved catalytic domain that uses NAD^+^ as the cofactor to transfer ADP-ribosyl group to the substrate protein (1, 2). This family of enzymes is responsible for the mono-ADP and poly-ADP ribosylation of cellular proteins resulting in modulation of their enzyme activity, cellular localization, and formation of multimeric protein complexes (3). Poly(ADP-ribosyl) polymerases (PARPs) are a subgroup of four enzymes (PARP1, PARP2, PARP5a, and PARP5b; PARP5a and PARP5b are also known as tankyrase1 [TNKS1] and tankyrase2 [TNKS2], respectively) that continue the reaction (PARylation) to generate long chains of linear and branched poly-ADP-ribose (PAR) on target proteins (4).

PARP1 is an abundant chromatin-associated nuclear protein that is important for genomic integrity (5). Activation of PARP1 is one of the earliest cellular responses to DNA damage. It leads to PARylation of histone and nuclear proteins and the consequent recruitment of DNA repair machinery to the site of damage (6). PARP1 is involved in repairing single-stranded DNA breaks making its inhibition an attractive strategy in oncology especially in those cancers that have defects in additional repair pathways (7). Inhibition of PARP1 results in the accumulation of DNA lesions, leading to double-stranded breaks during replication that are repaired by homologous recombination via breast cancer susceptibility proteins 1 & 2 (BRCA1 and BRCA2) (8). Thus, BRCA1/2 dysfunction sensitizes cells to cell-cycle arrest and apoptosis upon inhibition of PARP1 catalytic activity (9, 10). Hence, PARP1 has been one of the most intensely pursued drug discovery targets for the treatment of mutant BRCA1/2-driven cancers. Talazoparib, olaparib, niraparib, and rucaparib are Food and Drug Administration-approved PARP inhibitors, and veliparib is currently in late-stage clinical testing (11). Talazoparib and olaparib are approved for BRCA1/2 mutant breast cancers, whereas niraparib, olaparib, and rucaparib have been approved to treat ovarian cancer in different settings (12). A PARP inhibitor 2X-121 (formerly E7749) with activity against PARP1/2 and TNKS1/2 recently entered phase 2 clinical trial for the treatment of metastatic breast cancer, relapsed ovarian cancer, and pancreatic cancer (13).

Preclinically, talazoparib displays superior activity across tumor cell lines *in vitro* and *in vivo* where it can achieve response at much lower concentrations than other PARP inhibitors (14). Biochemically, PARP inhibitors have similar potency for their main cellular targets PARP1 and PARP2, which has led to the hypothesis that they act through two mechanisms to achieve activity. First, the catalytic inhibition of PARP1 in a homologous recombination-deficient background leading to double-stranded breaks and cell death. Second is the formation of a cytotoxic PARP–DNA complex (trapped complex) resulting in cell death (15). There is a strong correlation between the cellular activity of PARP inhibitors and their ability to stabilize the PARP–DNA complex. Recently, PARP1 inhibitors have been implicated in immunomodulatory function in cancer cells, activating cyclic GMP–AMP synthase–stimulator of interferon genes pathway, through their ability to stabilize PARP–DNA complex (16). Of the PARP inhibitors, talazoparib has the highest efficiency at stabilizing this complex (15, 17). Also, talazoparib shows more potent inhibitory activity against TNKS1/2 than other PARP inhibitors in biochemical assay (18).

TNKS1 and TNKS2 (collectively called TNKS) share 83% sequence identity overall, and their ART domain sequences are 89% identical. They play roles in DNA repair, telomere maintenance, and Wnt/β-catenin signaling (19). β-catenin levels, and hence the activity of Wnt/β-catenin pathway, are negatively regulated by a multiprotein destruction complex of which axin 1 and 2 are core components. Degradation of axins, induced by direct TNKS1/2-mediated PARylation, leads to decrease in the destruction complex activity, increase in β-catenin levels, and turning on of the Wnt/β-catenin pathway. Thus, TNKS activity promotes Wnt/β-catenin pathway (20), and TNKSs are potential drug targets for the treatment of cancers driven by Wnt/β-catenin pathway, most notably colorectal cancers (21). Though there is plethora of TNKS1/2 inhibitors described in preclinical studies (22, 23), a Food and Drug Administration–approved TNKS inhibitor has yet to enter the market.

In this work, we first apply chemical probes based on two PARP1 inhibitors, talazoparib and olaparib, in competition experiments in small cell lung cancer cell lysate and demonstrate that talazoparib at clinically relevant concentration interacts with PARP1 and TNKS1. Using surface plasmon resonance (SPR), we measured kinetic dissociation constants of several PARP inhibitors (talazoparib, olaparib, niraparib, and veliparib) to the catalytic domains of PARP1 and TNKS1. The results show that talazoparib possesses the strongest binding affinity to TNKS1, whereas its affinity for PARP1 is comparable to olaparib. We investigated the molecular bases of the differential affinities of these PARP inhibitors using hydrogen–deuterium exchange mass spectrometry (HDX–MS) and X-ray crystallography. Our study suggests that the inhibitor selectivity is largely driven by the interplay of protein and ligand dynamics, and the electrostatic nature of the ligand results in overt differences in the observed protein–ligand contacts, which in turn dictate their target selectivity.

## Results

### Talazoparib likely exhibits activity against PARP1/2 and TNKS1/2 at clinically relevant concentrations

**Figure 1:**
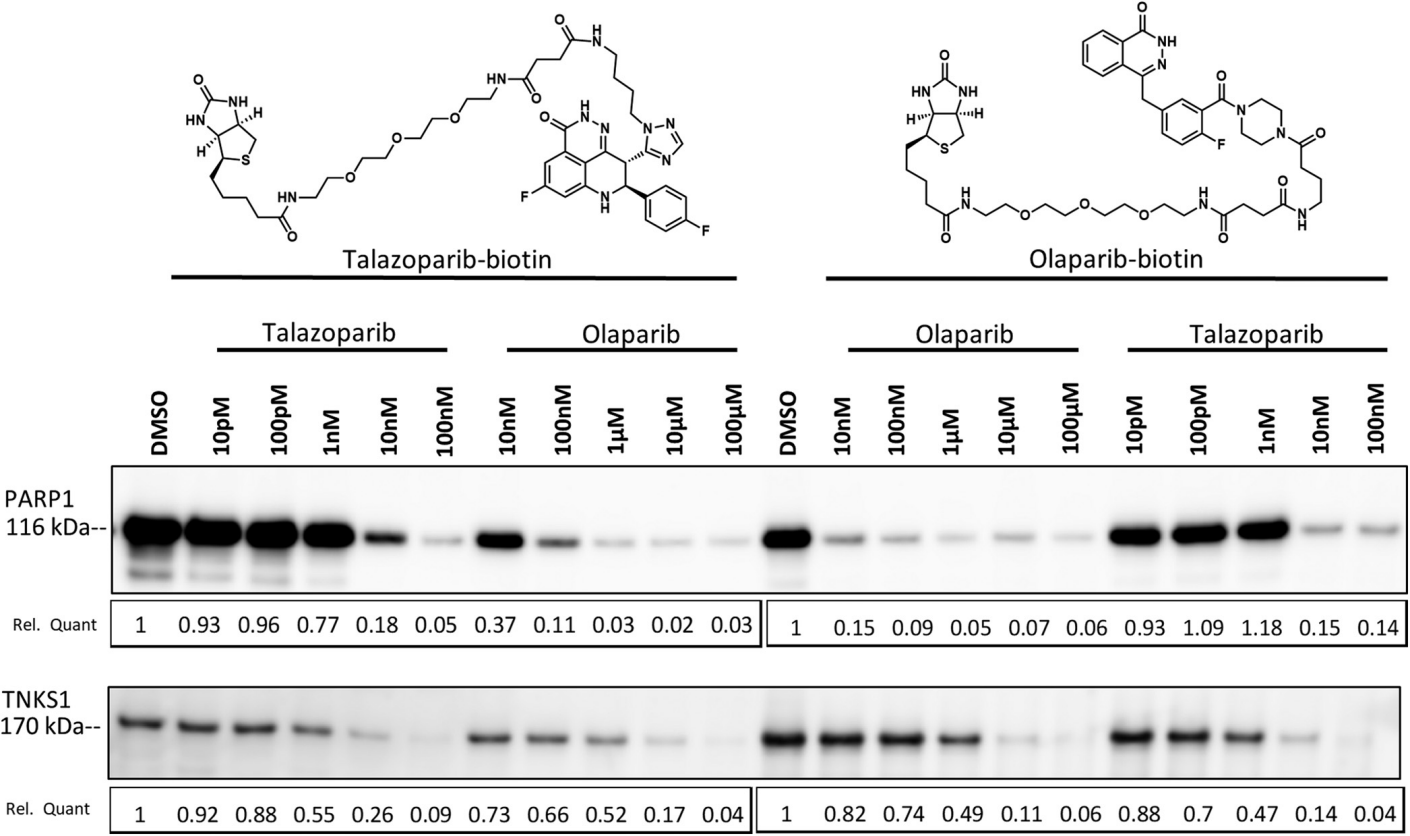
**Talazoparib binds to TNKS1 at a clinically relevant concentration in cultured cells.** NCI-H1048 cell lysate was pretreated with indicated concentrations of talazoparib or olaparib (30 min) prior to the addition of talazoparib– or olaparib–biotin probes (30 min). Proteins bound to streptavidin were released and subjected to Western blot analysis for either PARP1 (*top*) or TNKS1 (*bottom*). Relative quantitation (Rel Quant) was determined using ImageJ and is displayed below the plots. PARP1, poly(ADP-ribosyl) polymerase 1; TNKS1, tankyrase1.

Talazoparib and olaparib display similar biochemical potency on their main cellular targets (PARP1/2) and differential potency across the larger PARP family (17). To assess if these observations extend to endogenous cellular settings, we developed biotinylated chemical probes for talazoparib and olaparib and applied them in NCI-H1048 cellular lysate to competitively profile PARP1 or TNKS1 engagement with their corresponding parent inhibitors (Fig. 1). Olaparib and talazoparib exhibited similar ability to compete PARP1 binding to either chemical probe, whereas talazoparib uniquely exhibited an approximately 1000-fold greater ability to prevent binding to TNKS1 (Fig. 1, *right*). Taken together with the biochemical data, talazoparib and olaparib have equal potencies for their main cellular target PARP1, whereas talazoparib can more potently engage TNKS1. Given the free plasma concentration of ∼17 nM talazoparib at the clinical dose of 1 mg once a day (24), these data suggest that talazoparib would impair the activity of TNKS proteins at clinically relevant concentrations (23).

### Talazoparib has strong binding affinity for PARP1 and TNKS1 compared with other PARP inhibitors

Using SPR spectroscopy, we measured interaction kinetics of PARP1 inhibitors with the catalytic domain of PARP1 (residues 662–1011) and the ART domain of TNKS1 (residues 1104–1314). It demonstrated a clear dichotomy between talazoparib and three other inhibitors (Table 1; Fig. S1). In general, all inhibitors displayed high affinity for PARP1. However, talazoparib and olaparib bound to PARP1 with seven- to tenfold greater affinity than veliparib and niraparib, and this higher affinity was primarily driven by their relatively slow dissociation kinetics with dissociative half-life (*t*_1/2_) in hours compared with minutes. To this end, longer *t*_1/2_ of drug–target complexes has been shown to be intimately linked to drug efficacy (25, 26). Therefore, these data may help explain superior efficacy of talazoparib over other inhibitors. In addition, talazoparib distinctly bound to TNKS1 with an affinity that is not only two to three orders of magnitude higher than that of other inhibitors but also in the clinically relevant concentration range for TNKS1 engagement. Therefore, the TNKS1-binding data further differentiate talazoparib from other PARP1 inhibitors.

**Table 1:** Kinetics of PARP1 inhibitor binding to PARP1 and TNKS1^a^^,^^b^

Inhibitor	PARP1				TNKS1			
	*k*_on_ (M^−1^ s^−1^)	*k*_off_ (s^−1^)	*t*_1/2_ (min)	Kin. *K*_D_ (nM)	*k*_on_ (M^−1^ s^−1^)	*k*_off_ (s^−1^)	*t*_1/2_ (min)	Kin. *K*_D_ (nM)
Talazoparib	1.3 × 10^5^	7.3 × 10^−5^	158	0.6 ± 0.1	1.8 × 10^5^	2.6 × 10^−3^	4	14 ± 1
Olaparib	1.1 × 10^5^	8.8 × 10^−5^	131	0.8 ± 0.1	1.1 × 10^5^	1.7 × 10^−1^	0.1	1700 ± 6
Veliparib	7.5 × 10^5^	3.6 × 10^−3^	5	6.2 ± 3.5	3.1 × 10^3^	5.4 × 10^−2^	0.2	17,500 ± 300
Niraparib	3.4 × 10^5^	1.7 × 10^−3^	7	5.4 ± 1.4	2.5 × 10^4^	8.3 × 10^−1^	0.01	34,800 ± 540

a Binding kinetics measured in 25 mM Hepes, 150 mM NaCl, 5% glycerol, 0.5 mM TCEP, 2% DMSO, 0.02% Tween 20, and pH 7.4, at 25 °C.

b Each value in the table represents an average of two or more independent measurements.

### Interactions between the inhibitors and PARP1 are limited to the catalytic domain

**Figure 2:**
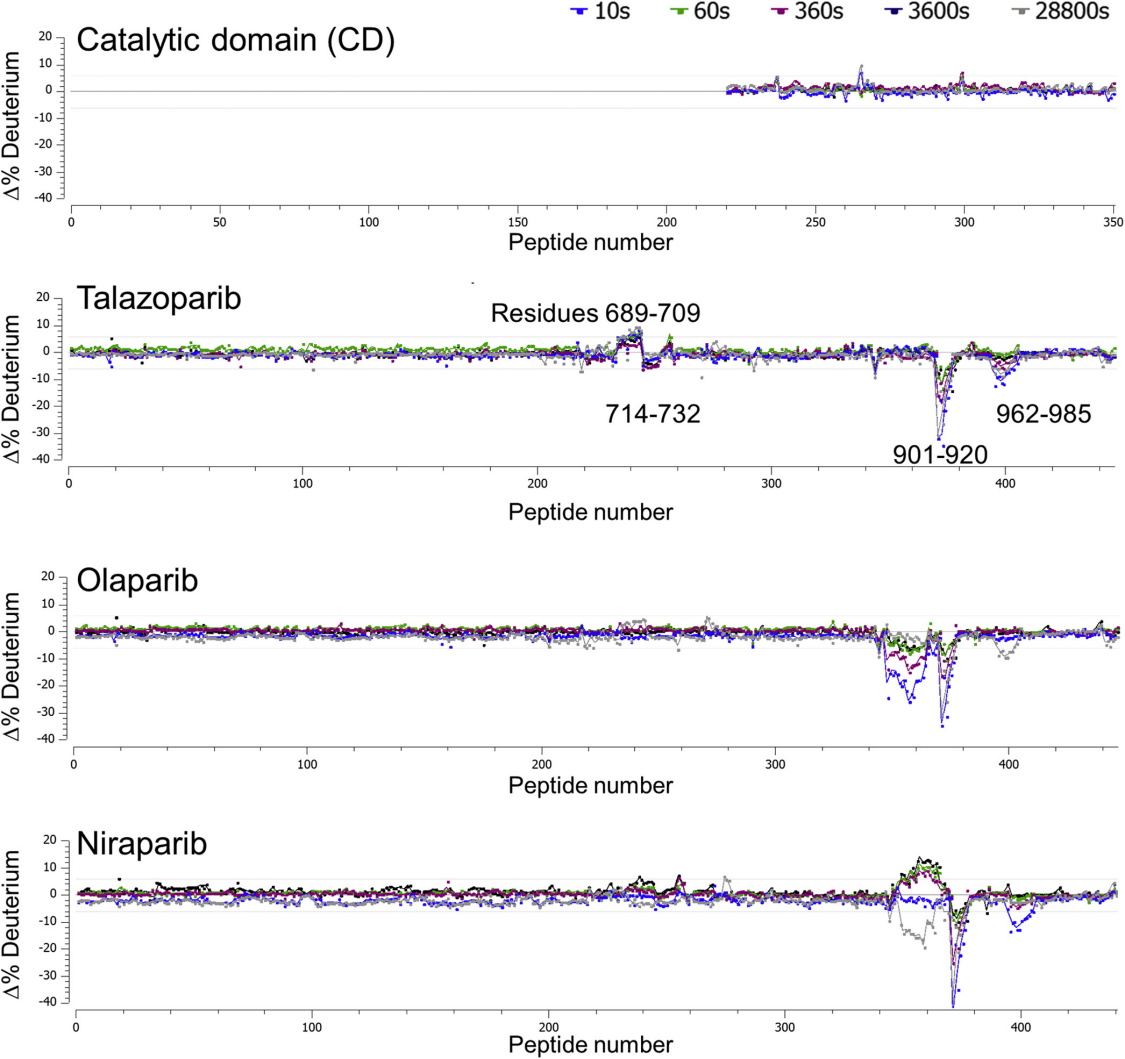
**Differential hydrogen–deuterium exchange profiles of poly(ADP-ribosyl) polymerase 1 (PARP1).** The *top panel* shows the profile for the isolated catalytic domain relative to the full-length PARP1. Subsequent panels show the perturbations in full-length PARP1 deuterium uptake with addition of various PARP inhibitors, all of which are localized within the catalytic domain.

The full-length PARP1 remains crystallographically intractable and structurally uncharacterized to date. As a result, we were limited to the structural characterization of the protein–ligand interactions using the isolated catalytic domain. In order to ascertain that the conclusions reached using the catalytic domain could be extended to the native enzyme, we first conducted the HDX–MS study of the full-length PARP1 and its isolated catalytic domain. The differential HDX–MS profile shown in the top panel of Figure 2 illustrates that the deuterium exchange for the catalytic domain remains the same irrespective of the context. This suggests that the catalytic domain can be treated as an isolated structural module that is not significantly influenced by the other domains.

Furthermore, HDX–MS study of the full-length PARP1 protein with talazoparib, olaparib, and niraparib suggested that the catalytic domain is the primary site of the protein–ligand interactions in the solution state (Fig. 2). In each instance, the change in the deuterium exchange profile of PARP1 upon the ligand recognition is limited to the catalytic domain of the enzyme. The most prominent features of the HDX residuals are restricted to the ART domain, residues 798 to 1014, though small localized (residues 689–709 and 714–732) changes in deuterium exchange profile within the helical domain are observed in the presence of talazoparib. This suggested that the isolated catalytic domain is an appropriate surrogate for the structural investigation of protein–ligand interactions. Reliable HDX–MS profile of PARP1 or TNKS1 in the presence of veliparib could not be collected.

Analogously, we intended to investigate the relationship between the TNKS1 ART domain with the rest of the protein as well as the HDX signature of the protein–ligand interaction in the context of the full-length TNKS1. However, full-length TNKS1 showed poor solution behavior during purification; heavy aggregation and precipitation thwarted comparative HDX–MS characterization of the PARP inhibitor panel. However, we were able to characterize the TNKS1 ART domain interactions with talazoparib, olaparib, and niraparib using HDX–MS (Fig. S2). The results showed weaker perturbation of the deuterium exchange profile in the presence of ligand relative to what was observed with PARP1. We believe that the weaker protein–ligand interaction signature of TNKS1 ART domain is the result of faster off-rates and the consequent shorter half-lives of the complexes.

### Structural characterization of protein–ligand interactions

We determined crystal structures of the catalytic domain of PARP1 and the ART domain of TNKS1 in the absence and the presence of the ligands (Tables 2 and 3). The crystals belonged to multiple different crystal forms with multiple molecules in the asymmetric unit, providing valuable insights into the protein dynamics. It is also noteworthy that TNKS1 crystal structures reported here preserve the ART domain dimer recently proposed to be of biological significance (27).

**Table 2:** Data collection and refinement statistics for apo-PARP1 structure and its complexes with inhibitors

Data collection statistics (PDB ID)	Apo PARP1 (7KK2)	Talazoparib (7KK3)	Olaparib (7KK4)	Niraparib (7KK5)	Veliparib (7KK6)
Wavelength (Å)	1.0	1.0	1.0	1.0	1.0
Resolution	78.9–1.7	86.1–2.1	88.9–2.0	83.9–1.7	60.78–2.06
Space group	P2_1_2_1_2_1_	P2_1_2_1_2_1_	C2	P2_1_2_1_2_1_	P2_1_2_1_2_1_
Unit cell	48.1, 91.6, 162.8	104.5, 107.7, 143.5	172.8, 44.5, 111.9, *β* = 127.4	104.0, 108.5, 141.8	48.5, 91.4, 162.8
Total number of reflections	357,785	530,258	109,151	1042,187	180,476
Unique reflections	58,284	79,507	33,313	159,983	29,062
Multiplicity	6.1	6.7	3.3	6.5	6.2
Completeness (%), spherical^a^	74.4 (18.5)	79.6 (18.8)	68.2 (13.3)	92.2 (38.9)	63.2 (10.8)
Completeness (%), ellipsoidal^a^	89.9 (46.5)	94.8 (62.1)	91.7 (61.3)	96.1 (56.7)	91.3 (55.7)
Mean I/σ(I)^a^	13.1 (1.5)	9.8 (1.5)	11.4 (1.7)	15.8 (1.4)	6.4 (1.4)
Rmerge^a^	0.06 (0.906)	0.112 (1.431)	0.057 (0.815)	0.064 (1.13)	0.213 (1.377)
Rpim^a^	0.033 (0.507)	0.047 (0.592)	0.038 (0.537)	0.027 (0.528)	0.095 (0.6)
CC1/2^a^	0.998 (0.50)	0.997 (0.492)	0.998 (0.618)	0.999 (0.536)	0.981 (0.388)
Refinement statistics					
Reflections used	58,341	79,520	33,314	160,088	29,062
Reflections used for *R*_free_	2833	4066	1499	7931	1383
*R*	0.204	0.215	0.217	0.206	0.206
*R*_free_	0.2379	0.2592	0.266	0.238	0.271
RMS (bonds) (Å)	0.008	0.01	0.01	0.01	0.01
RMS (angles)	0.99	1.14	1.2	1.05	1.22
Ramachandran outliers (%)	0	0	0	0	0.2
Average B-factor (Å^2^)	31.8	60.7	52.5	38.6	42.5
MolProbity clashscore (percentile)	2.7 (99)	2.56 (99)	3.85 (99)	1.19 (99)	4.04 (99)

a The values in parenthesis are for the highest resolution shell.

**Table 3:** Data collection and refinement statistics for apo-PARP1 structure and its complexes with inhibitors

Data collection statistics (PDB ID)	Apo TNKS1 (7KKM)	Talazoparib (7KKN)	Olaparib (7KKO)	Niraparib (7KKP)	Veliparib (7KKQ)
Wavelength (Å)	1.0	1.0	1.0	1.0	1.0
Resolution	80.5–1.6	83.3–1.5	57.1–1.6	75.9–1.4	79.6–1.6
Space group	P2_1_	P2_1_	C222_1_	P2_1_2_1_2_1_	P2_1_2_1_2_1_
Unit cell	49.0, 114.9, 80.5, *β* = 91.5	48.7, 113.9, 83.4, *β* = 91.0	79.3, 90.4, 199.8	43.4, 74.1, 151.8	76.2, 84.3, 159.1
Total number of reflections	293,492	342,300	482,274	460,475	703,070
Unique reflections	91,574	109,090	81,856	75,704	110,251
Multiplicity	3.2	3.1	5.9	6.1	6.4
Completeness (%), spherical^a^	75.0 (17.6)	79.0 (26.4)	81.4 (28.1)	81.4 (24.5)	80.3 (22.9)
Completeness (%), ellipsoidal^a^	89.2 (47.3)	87.1 (46.8)	88.6 (58.9)	92.7 (52.3)	92.8 (51.8)
Mean I/σ(I)^a^	14.8 (1.5)	13.8 (1.5)	10.2 (1.5)	15.6 (1.5)	19.1 (1.8)
Rmerge^a^	0.055 (0.645)	0.051 (0.538)	0.094 (0.978)	0.059 (0.799)	0.047 (0.789)
Rpim^a^	0.036 (0.477)	0.033 (0.456)	0.042 (0.536)	0.025 (0.439)	0.02 (0.502)
CC1/2^a^	0.999 (0.611)	0.998 (0.606)	0.996 (0.453)	0.999 (0.538)	0.999 (0.786)
Refinement statistics					
Reflections used	91,556	110,073	81,880	75,846	110,379
Reflections used for *R*_free_	4640	5401	4031	3700	5532
*R*	0.195	0.184	0.194	0.187	0.204
*R*_free_	0.233	0.215	0.221	0.208	0.23
RMS (bonds) (Å)	0.01	0.01	0.01	0.01	0.01
RMS (angles)	1.01	1.01	1.06	1.01	1.05
Ramachandran outliers (%)	0	0	0.2	0	0.1
Average B-factor (Å^2^)	27.2	22.7	29.8	20.9	30.2
MolProbity clashscore (percentile)	1.18 (99)	2.08 (99)	1.46 (99)	1.04 (0.99)	1.86 (99)

a The values in parenthesis are for the highest resolution shell.

As details of PARP1 catalytic domain and TNKS1 ART domain have been described (28, 29), we focus only on the inhibitor-binding aspect of these proteins.

#### The helical domain and donor loop distinguish the ligand-binding sites of the two enzymes

**Figure 3:**
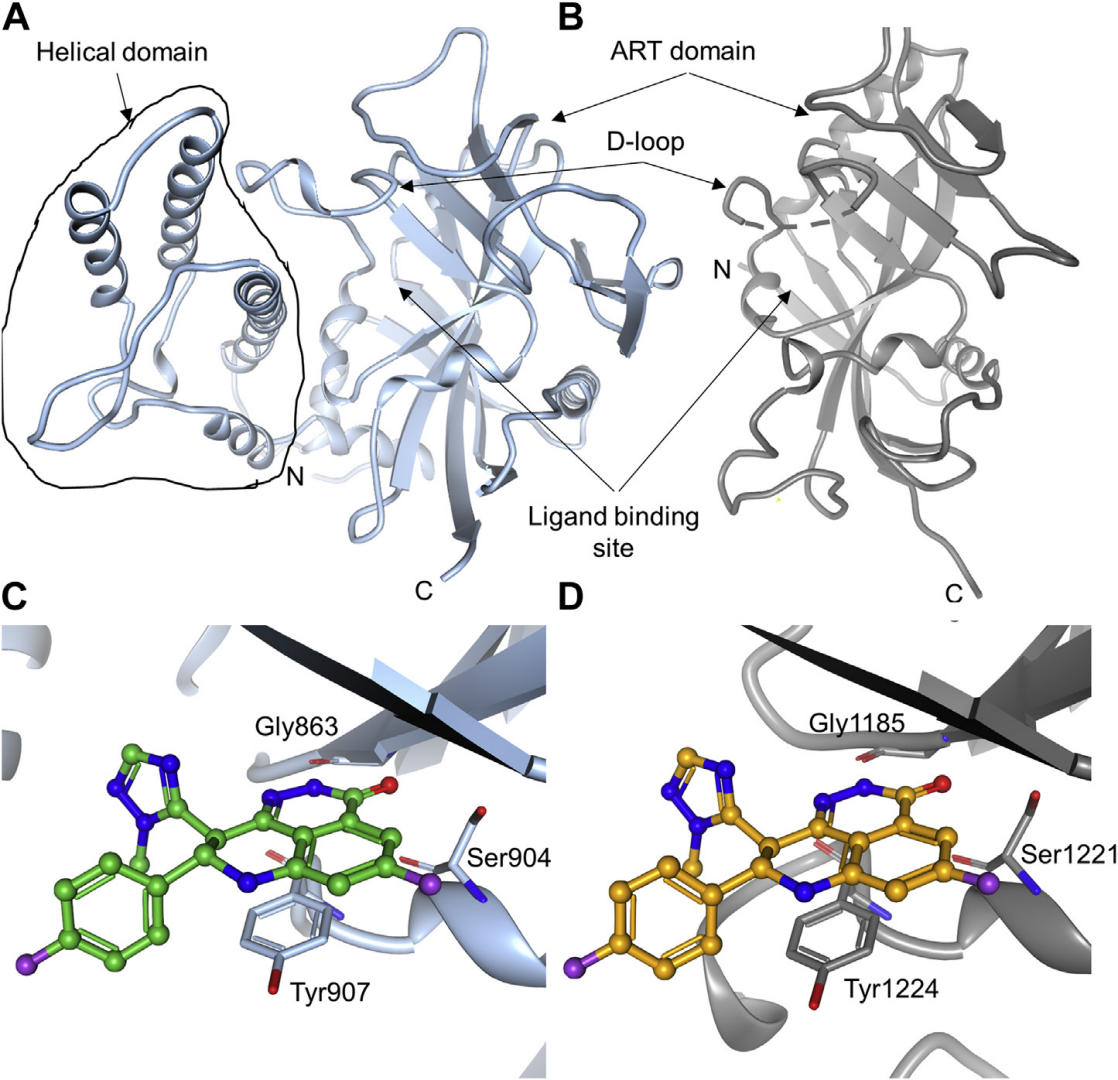
**Overall structures of the catalytic domain of poly(ADP-ribosyl) polymerase 1 (PARP1) and the ART domain of tankyrase1 (TNKS1) and similarities in the ligand-binding sites.***A*, PARP1 catalytic domain is composed of the helical domain and ART domain. *B*, TNKS1 ART domain. The broken line shows the disordered region of TNKS1 D-loop that is not modeled due to the absence of electron density. Recognition of talazoparib by (*C*) PARP1 and (*D*) TNKS1 is similar in the two proteins. ART, ADP-ribosyltransferase; D-loop, donor loop.

The catalytic domain of PARP1 includes a helical domain followed by the prototypical ART domain (Fig. 3*A*). Structurally, the helical domain is placed adjacent to the NAD^+^/ligand-binding site and limits its exposure to solvent. TNKS1, on the other hand, does not contain the helical domain, and its NAD^+^/ligand-binding site is seen to be more solvent exposed in isolated ART domain structures (Fig. 3*B*). This could be one contributing factor for higher dissociation rate and kinetic dissociation constants, for inhibitor complexes with TNKS1 relative to PARP1 complexes (Table 1). The second difference is in the nature of the donor loop (D-loop); in PARP1, it is longer and more rigid than in TNKS1. PARP1 D-loop has three proline residues in its sequence, which likely contribute to its rigidity. In addition, interaction with the helical domain might make the D-loop structurally constrained (Fig. S3). The absence of both these features might contribute to the observed dynamic nature of TNKS1 D-loop (Fig. 3*B*). The crystal structure of apo-PARP1 catalytic domain shows that the ligand-binding site is largely preformed, and the inhibitors can be accommodated with minimal structural rearrangement. In contrast, in apo-TNKS1, the D-loop is dynamic, potentially occluding the ligand-binding site, and must remodel for ligand recognition (Figs. 3*B* and S3). Perhaps consistent with this logic, ligand recognition induces a rotamer change in the Phe1188 side chain of TNKS1. The associated energetic cost of these conformational changes is likely reflected in the larger dissociation constants of TNKS1 complexes relative to PARP1 complexes.

#### All four inhibitors share some aspects of PARP1 or TNKS1 recognition

All four PARP inhibitors target the nicotinamide-binding pocket of both enzymes as expected since they were designed based on the nicotinamide-like pharmacophore. Pyridazinone moiety of talazoparib and olaparib and the amide groups of niraparib and veliparib mimic the amide group of nicotinamide. Inhibitors stack on Tyr907 in PARP1 and make conserved hydrogen bonding interactions with backbone nitrogen and carbonyl oxygen of Gly863 and the sidechain hydroxyl of Ser904 (Fig 3*C*). Likewise, in TNKS1, ligands stack on Tyr1224 and make hydrogen bonding interactions with the corresponding backbone atoms of Gly1185 and the sidechain hydroxyl of Ser1221 (Fig. 3*D*). These structural observations are consistent with the PARP1 HDX–MS data, which show the biggest change in the HDX profile upon ligand recognition in the polypeptide spanning 901 to 920.

#### Higher affinity of talazoparib for PARP1 relative to TNKS1 is due to the differences in the ligand-binding sites

**Figure 4:**
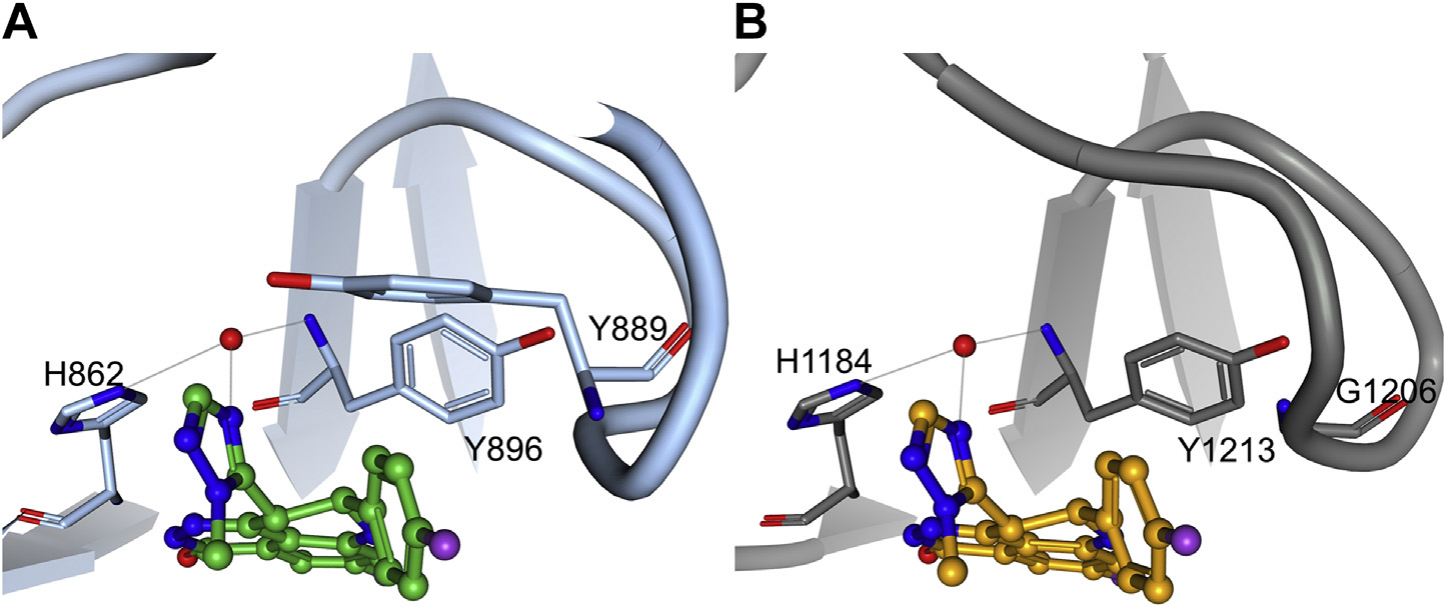
**Talazoparib makes a favorable interaction with the structurally conserved water molecule in poly(ADP-ribosyl) polymerase 1 (PARP1) and tankyrase1 (TNKS1) catalytic domains.** The structural water molecule is shown as *red sphere*. *A*, PARP1 recognition of talazoparib. *B*, TNKS1 recognition of talazoparib.

PARP1–talazoparib complex structure is similar to the previously reported structure (30). The TNKS1–talazoparib complex structure clearly reveals conformational change in the protein D-loop upon ligand recognition (Fig. S3). The molecular mechanism of recognition is largely similar in the two proteins but for one difference (Figs. 4 and S4). There is a single substitution of Tyr889 in PARP1 with Gly1206 in TNKS1 in the D-loop. Tyr889 of PARP1 is seen offering van der Waals interaction to the bound talazoparib. In addition, Tyr889 is also poised to make edge-to-face (pi) interaction to the fluorophenyl group of talazoparib. These additional stabilizing interactions are absent in the TNKS1 complex (Fig. 4). Thus, the stronger affinity of talazoparib for PARP1 is likely driven by the well-ordered and enclosed ligand-binding site by virtue of the structured D-loop and the presence of the helical domain.

#### Interaction with a structural water and the rigid ligand structure makes talazoparib a superior inhibitor of PARP1 and TNKS1

In addition, talazoparib makes a specific interaction with a conserved water molecule. In all structures reported here, a structural water molecule is observed (except for the olaparib-bound structures, where it is displaced by the carbonyl oxygen of the ligand; see later). This water molecule makes hydrogen bonding interactions with the imidazole sidechain of the catalytic His862 and the backbone amide nitrogen of Tyr896 in PARP1 and analogously with His1184 and Tyr1213 of TNKS1 (Fig. 2). Talazoparib uniquely makes a specific interaction with this water molecule through its triazole nitrogen atom, which serves as a hydrogen bond acceptor. This feature could afford talazoparib its superior binding affinity for both PARP1 and TNKS1 relative to the other inhibitors. Also contributing to the superior binding affinity of talazoparib is its rather rigid structure with two rotatable bonds, which is recognized in its low-energy conformation by both proteins, unlike in the cases of olaparib and niraparib (see later).

#### Olaparib and niraparib adopt strained conformations for TNKS1 recognition

**Figure 5:**
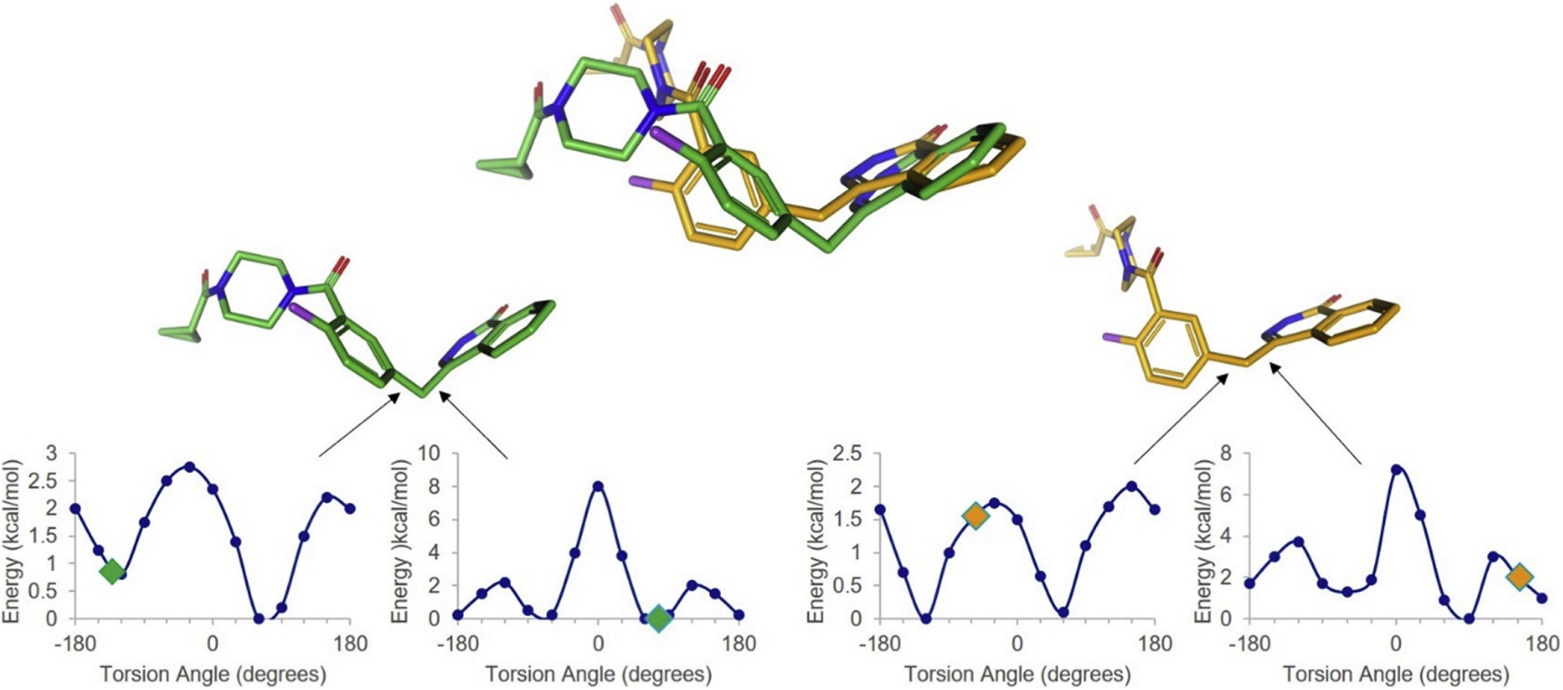
**Olaparib energetics when bound to PARP1 or TNKS1.** The two olaparib conformational states (bound to PARP1 in *green* and bound to TNKS1 in *orange*) are superposed on top. The energy profiles of two torsion angles are shown, which have lower energy in the PARP1-bound conformation (*green*, *left*), relative to the TNKS1-bound conformation (*orange*, *right*). The torsional energies of the other rotatable bonds are very similar in the two conformers. PARP1, poly(ADP-ribosyl) polymerase 1; TNKS1, tankyrase1.

Olaparib is the largest PARP1 inhibitor studied here and is the only one that extends into the adenine-binding pocket of PARP1/TNKS1. It adopts different conformations for the recognition of PARP1 and TNKS1 (Fig. 5), despite making similar interactions with the two proteins (Fig. S5). In order to evaluate the energetics of the bound inhibitor conformation, we performed the torsion strain analysis (31) of each of the rotatable torsion angles of the ligand in its PARP1- and TNKS1-bound conformations. Overall, the PARP1-bound conformation of olaparib adopts lower energy torsional states than the ones adopted by TNKS1-complexed conformation (Fig. 5). As a result, we postulate that the TNKS1-bound olaparib is a higher energy state than the PARP1-bound conformation, which compromises the binding affinity of olaparib for TNKS1. Further analysis suggests why the lower energy PARP1-bound conformation of olaparib may be incompatible with TNKS1 recognition (Fig. S5*C*).

When the PARP1-complexed conformation of olaparib is modeled into TNKS1 structure, severe steric conflicts with the D-loop are observed. Some of these conflicts can only be alleviated by major remodeling of the D-loop, specifically the base of the loop consisting of Glu1199 and Arg1200 (Fig. S6). However, both these residues contribute important interactions to the TNKS1 dimer formation (27) and are likely not flexible enough as evidenced by low crystallographic B-factors and conserved rotamers across structures. Thus, the changes required in the D-loop conformation may not be energetically favorable.

**Figure 6:**
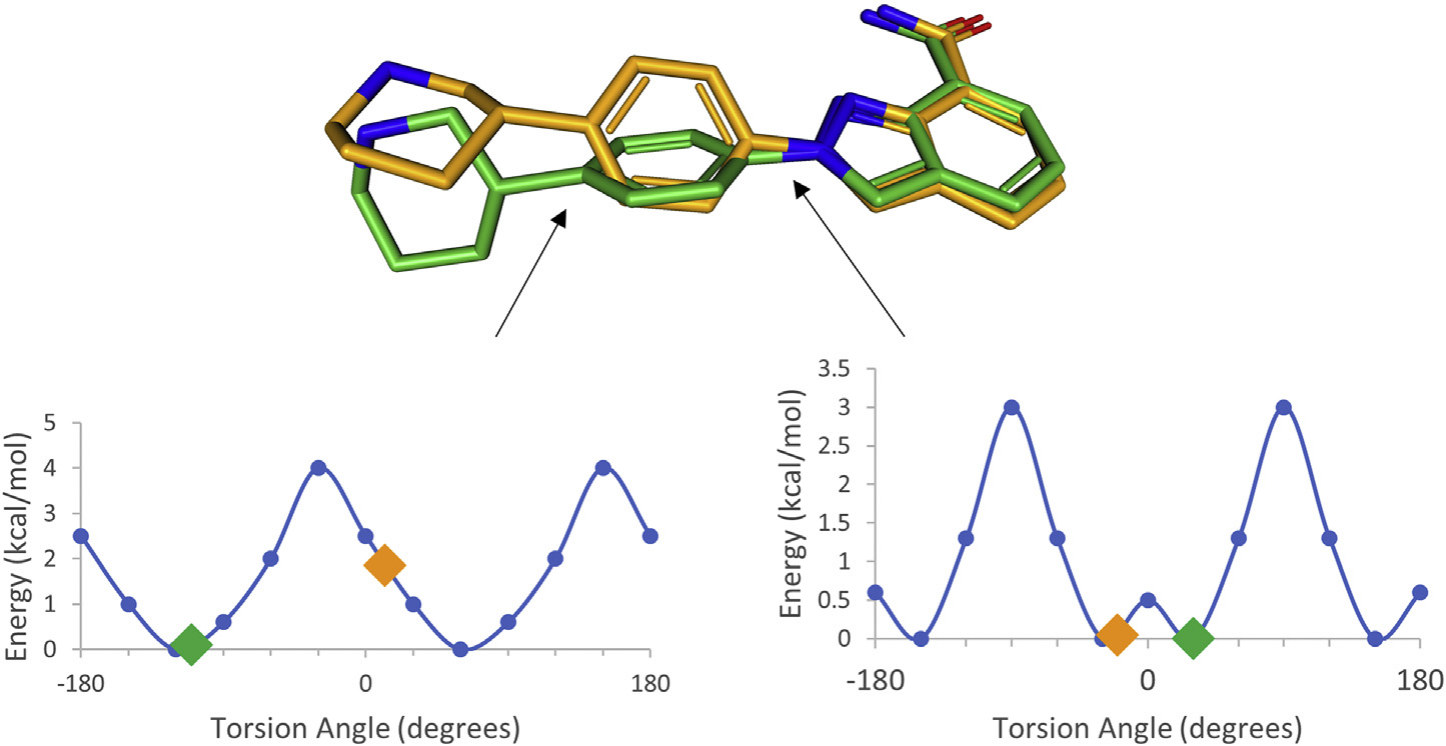
**Niraparib energetics when bound to PARP1 (*green*) or TNKS1 (*orange*).** The two niraparib conformational states (bound to PARP1 in *green* and bound to TNKS1 in *orange*) are superposed on *top*. The energy profiles of two torsion angles are shown at the *bottom*. The torsion angle between the phenyl ring and the piperidine is clearly more favorable energetically in the PARP1-bound conformation (*green*, *left*), relative to the TNKS1-bound conformation (*orange*, *right*). The torsional energies of the other rotatable bonds are very similar in the two conformers. PARP1, poly(ADP-ribosyl) polymerase 1; TNKS1, tankyrase1.

PARP1–niraparib structure is similar to the previously reported cocrystal structure (18). TNKS1–niraparib structure has two molecules in the asymmetric unit, and they show different D-loop conformations. In one of the protein molecules, the D-loop is significantly raised relative to the ligand-binding site, offering minimal ligand-binding contacts. Consequently, the piperidine ring of niraparib has no interpretable electron density, and it is not modeled. In the second molecule, the D-loop is lowered and offers some stabilizing interactions so that the entire ligand could be modeled (Fig. S7). This variation among molecules in the asymmetric unit of the same crystal structure suggests lack of intimate stabilizing interactions between the protein and the ligand. Furthermore, we analyzed the torsion energy profile of the rotatable bonds of the ordered niraparib molecule. The torsion angle of the rotatable bond connecting the phenyl ring with the piperidine ring is at a significantly higher energy state than the corresponding torsion in PARP1–niraparib structure (Fig. 6). When the lower energy PARP1-bound conformation of niraparib is modeled into the TNKS1 structure, steric conflicts with Ile1228 are observed (Fig. S7). Thus, like olaparib, the small molecule conformational energetics contribute to the weaker binding potency of niraparib to TNKS1.

#### Basic groups on niraparib and veliparib provide complementary electrostatic potential to acidic sidechains from the helical domain of PARP1

**Figure 7:**
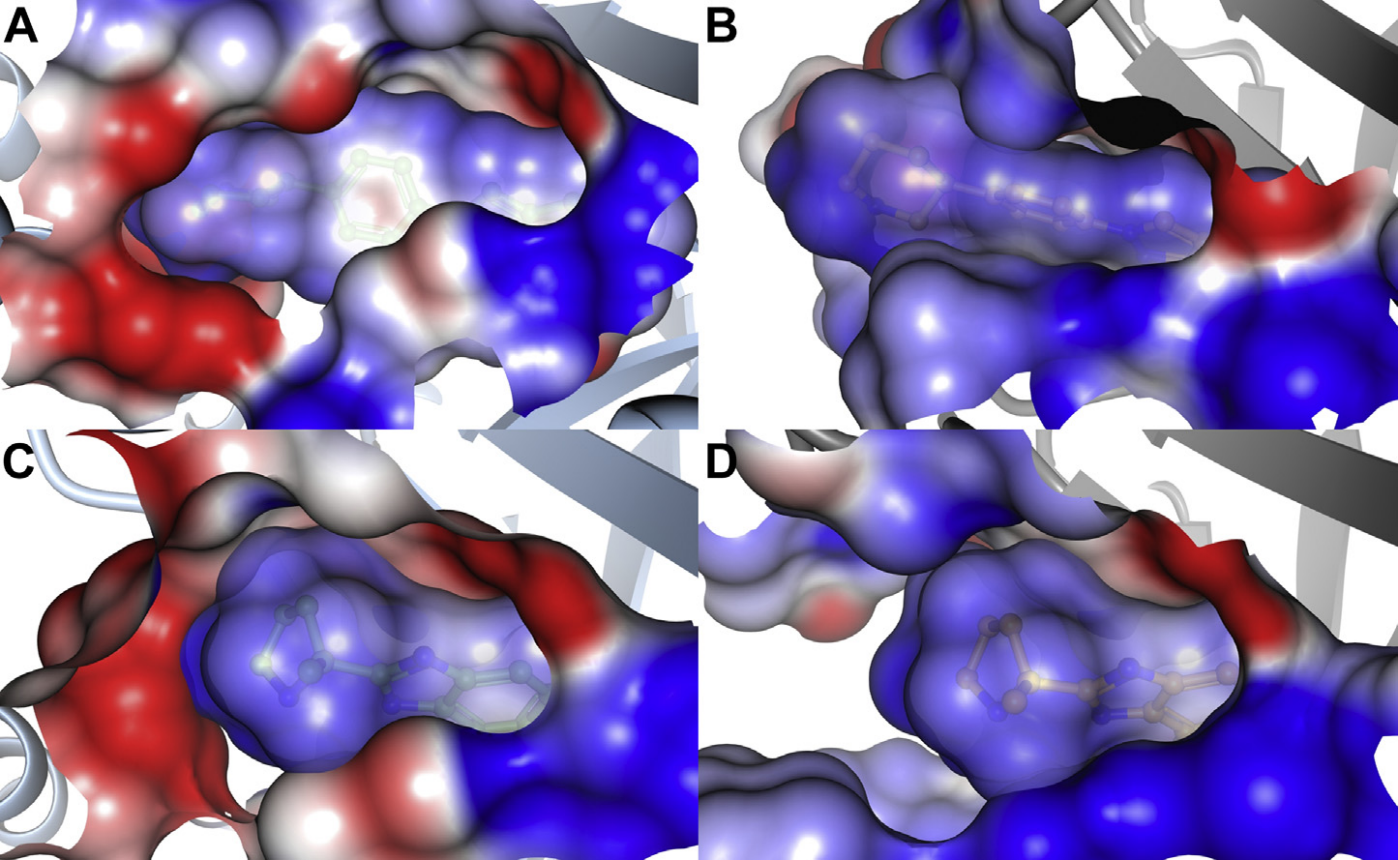
**PARP1-binding site offers better electrostatic surface complementarity for niraparib and veliparib recognition than TNKS1-binding site.** Electrostatic surface representation (*blue* denotes positive and *red* negative electrostatic surface) of (*A*) PARP1-niraparib, (*B*) TNKS1-niraparib, (*C*) PARP1-veliparib, and (*D*) TNKS1–veliparib complexes. PARP1, poly(ADP-ribosyl) polymerase 1; TNKS1, tankyrase1.

Niraparib and veliparib have piperidine and pyrrolidene ring, respectively, oriented toward the helical domain of PARP1. Figure S8 illustrates the compound structures and the electrostatic surface representation of the ligands and of the ligand-binding sites of the proteins. The basic nitrogen of the piperidine ring of niraparib makes a hydrogen bonding interaction with Glu766 from the helical domain of PARP1 (Fig. S7). In addition, the piperidine nitrogen coordinates a water molecule that makes network of interactions with the protein backbone as well as surrounding water molecules. On the other hand, there is poor charge complementarity between the TNKS1-binding site and the piperidine of the ligand (unlike PARP1 where acidic groups from the helical domain create favorable electrostatic environment) (Fig. 7).

Veliparib is the smallest, and likely the most rigid, of the ligands studied here. Its mode of recognition by PARP1 and TNKS1 resemble each other (Fig. S9), and the ligand conformational energetics are comparable. However, veliparib is a basic molecule with a formal positive charge on the pyrrolidine ring. The pyrrolidine ring nitrogen atom is within the striking distance of ion-pair interaction with Glu763 from the PARP1 helical domain. However, there is not an equivalent interaction in TNKS1. On the contrary, there is very poor electrostatic complementarity between veliparib and the binding site of TNKS1, both being electropositive (Fig. 7). This could contribute to very high off-rates, slow on-rates, and large dissociation constants of verliparib-TNKS1 complex.

Further we interrogated the role of electrostatics in the PARP1 ligand recognition by conducting additional SPR experiments at a higher salt concentration (0.5 M) (Table S1). A dominant role for electrostatics would be characterized by an increase in the measured dissociation constants at higher salt concentration. However, we saw the opposite effect (Tables 1 and S1) implying that hydrophobic interactions played significant role in ligand recognition (32). However, the effect was smaller for veliparib and niraparib (which are more basic) than talazoparib and olaparib, underscoring potentially bigger role played by electrostatics in niraparib and veliparib recognition.

Thus, strong binding affinity of talazoparib for both PARP1 and TNKS1 relative to the other inhibitors studied here could be attributed to its rigid structure that is unencumbered by conformational energy penalty in either of the complexes, its ability to make stabilizing interaction with structural water in both enzymes and the lack of overtly electropositive nature. The stronger binding affinity of talazoparib for PARP1 relative to TNKS1 is most likely because of the presence of the helical domain in PARP1 and the differences in the D-loop of the two enzymes in terms of the amino acid sequence and its conformational dynamics. Selectivity of olaparib for PARP1 over TNKS1 is largely because of conformational energetics of the ligand. Niraparib selectivity can be explained based on its conformational energetics as well as the electrostatic effect, whereas veliparib's overtly basic nature might be responsible for its selectivity for PARP1.

## Discussion

At the onset of this investigation, we explored the relationship of the PARP1 catalytic domain with the rest of the protein. It is evident that the structure of the catalytic domain is not influenced by the rest of PARP1 to the extent that we could study it in solution state using HDX–MS. This is not surprising given that numerous available structures of the catalytic domain, including the one spanning multiple PARP1 domains (33), suggest that it is largely a stable structure. Likewise, the ligand binding is localized to the catalytic domain.

The stability of the protein–ligand interactions depends on multitude of factors, including the protein and ligand dynamics, the energetic cost of conformational adaptations and solvation, besides the strength of direct interactions (*viz*. specific ion pairs and hydrogen bonding, nonspecific van der Waals interactions and water-mediated contacts). Since the study here pertains to the selectivity of the same ligands for two different enzymes, we can ignore the solvation and dynamics of the isolated ligands. Whereas previous studies have suggested that the molecular weight and hydrophobicity of various PARP inhibitors were the primary determinants of their selectivity profile (34), we observe here that the electrostatic potential of the ligand may also be a factor. The acidic sidechains from the helical domain contribute partial negative electrostatic potential to the PARP1 inhibitor-binding pocket, differentiating itself from that in TNKS1. Thus, compounds with basic groups oriented toward the helical domain would show selectivity for PARP1 as we see in the case of niraparib and veliparib. Another inhibitor not included in this study, PJ34, has a terminal tertiary amine group and has over 25-fold lower IC50 for PARP1 than that for TNKS1 (18). Conversely, an acidic group would be predicted to prefer TNKS recognition over PARP1. If the electrostatics were the dominant driver of the ligand recognition, we could have potentially expected to see increase in the dissociation constant at higher salt concentration in our SPR experiment. However, higher salt concentration also increases hydrophobic interactions and viscosity, which make substantial contribution to the ligand recognition (35). Nevertheless, the increase in the binding affinity at higher salt concentration was smaller for niraparib and veliparib than for talazoparib and olaparib, supporting the inference that the electrostatics play significant role in niraparib and veliparib recognition. Also unique to the present study is the interplay of the ligand strain and protein flexibility as one of the key contributors to the inhibitor selectivity. Using computational tools to evaluate the strain in the observed ligand conformation (31), olaparib and niraparib clearly show strained conformations in complex with TNKS1. Conclusions about protein dynamics and flexibility, on the other hand, can be derived from the solution-state HDX studies and the wealth of structural data.

As has been noted before, there is a fundamental difference between the D-loops of PARP1 and TNKS1 (34). Though the PARP1 D-loop is longer than that in TNKS1, it also has three proline residues that could impart greater rigidity. In addition, observed contacts and coordinated motion between the helical domain and the D-loop suggest that the PARP1 D-loop is restrained. The TNKS1 D-loop on the other hand is shorter, relatively flexible, and shows multiple conformations of its glycine-rich region (residues 1201–1211). Nevertheless, we conjecture that the base of the loop is significantly more rigid because it plays a pivotal role in dimerization. Glu1199 and Arg1200 at the base of the D-loop are involved in ion pair interactions with charged sidechains of the dimeric partner. This may limit the conformational space accessible to the D-loop. The net outcome is a smaller and more restrictive ligand-binding site in TNKS1 relative to that in PARP1. As a result, TNKS1 site can potentially recognize limited number of chemical conformations, which for numerous ligands may be higher energy/low abundance states. The crystal structures suggest that each inhibitor influences conformation of the flexible region of TNKS1 D-loop, some of which may be more favorable to chemical engagement than others.

The selectivity of therapeutic inhibitors contributes to the extent of off-target toxicities. Alopecia is one such frequent side effect (12). In case of PARP1 inhibitors, it could be due to the potent DNA-trapping ability of the inhibitor and the consequent cytotoxicity to the stem cells. However, given the well-established role of the Wnt/β-catenin pathway in hair progenitor and niche maintenance (33) and the role of TNKS enzymes in regulating this pathway, we cannot rule out that PARP inhibitors also impact this pathway in the hair follicle. Our conclusions based on the current inhibitors could help design the next generation of more selective PARP1 and TNKS1 inhibitors and identify additional therapeutic opportunities that exploit the polypharmacology of PARP inhibitors.

## Experimental procedures

### Cell line maintenance

The small cell lung cancer NCI-H1048 cells were originally obtained from American Type Culture Collection (CRL-5853) and thawed and recovered in the suggested hydrocortisone, insulin, transferrin, estrogen, and selenium medium supplemented with 5% fetal bovine serum. Cells were then gradually converted into RPMI medium supplemented with 10% fetal bovine serum (Gibco 26140079) and 1% penicillin–streptomycin (Gibco 1514122) for experiment consistency. Cells were maintained for a couple of passages before plating five 15-cm dishes at a 0.5 M/ml density in 48 ml of media the day before the experiment.

### Cell lysate preparation

The small cell lung cancer NCI-H1048 cells were washed three times with PBS (Gibco 10010023) and scrapped with 500 μl of cold prepared lysis buffer (immunoprecipitation lysing buffer [Pierce 87787] with protease and phosphatase inhibitor cocktail [Cell signaling 5872S] and 250 μg poly(ADP-ribose) glycohydrolase inhibitor PDD00017273 [Sigma SML1781-5mgs]) for 15 min on ice. Lysate was then spun at 1500 rpm for 3 min, and the soluble fraction was transferred to a new tube on ice. Protein lysate concentration was measured with BCA Protein Assay Kit (Pierce 23227) according to manufacturer's protocol.

### PARP inhibitor chemical probe pulldown

For each pulldown, 25 μl of magnetic streptavidin bead slurry (Pierce 8816) was washed in a 1.5-ml tube according to manufacturer's protocol. PARP inhibitor-biotinylated compounds (talazoparib: 1.7 μl from 10 mM stock and olaparib: 8.75 μl from 100 μM) were conjugated to beads for 1 h rotating at room temperature. Conjugated beads were washed three times with wash buffer (50 mM Hepes [Fisher Scientific AAJ60712AK], 150 mM NaCl [Invitrogen, AM9759], and 1% nonyl phenoxypolyethoxylethanol [Fisher Scientific NC9168253]) rotating for 3 min. All PARP inhibitor pulldowns were from 1 mg/ml of protein lysate. Lysate for pulldowns was first incubated with dimethyl sulfoxide (DMSO) or PARP inhibitors talazoparib or olaparib in defined increasing concentration rotating for 30 min at 4 °C. Lysate samples were then added to conjugated beads as defined and rotated for 30 min at 4 °C. All beads were washed three times with wash buffer rotating for 3 min. Bead samples were resuspended in 1× SDS loading dye, and bound proteins were eluted from bead by boiling for 10 min.

### Western blot

Bead protein elutions were separated on 4 to 12% protein gel (3450125 BioRad) and wet transferred to a polyvinylidene difluoride membrane (17001919 BioRad) for 30 min at 100 V in 20% methanol Tris–glycine running buffer. Membranes were blocked for 1 h at room temperature and stained overnight at 4 °C with PARP1 (Invitrogen PA5-34802; 1:500 dilution) and TNKS (Bethyl Labs A302-399A; 1:1000 dilution) antibodies. Membranes were washed with 1× tris-buffered saline (Teknova T9511) and then stained with anti-rabbit horseradish peroxidase–conjugated secondary antibody (Cell Signaling 7074) for 1 h at room temperature. Chemiluminescence was detected with Super Signal West Femto (Thermo Fisher 34095) and imaged with a ChemiDoc system.

### Gel quantification

Western blot protein bands were quantified using BioRad Image Lab as suggested by software's user manual.

### Measurement of binding kinetics using SPR

#### Chip functionalization

Biacore S200 instrument was desorbed and loaded with a new Series S Sensor Chip SA at 10 °C. The recombinant biotinylated PARP1 (amino acids: 662–1011) or biotinylated TNKS1 protein (amino acids: 1104–1314) was diluted to 50 μg/ml with assay buffer (50 mM Hepes, 150 mM NaCl, 0.5 mM tris(2-carboxyethyl)phosphine [TCEP], 5% glycerol, 0.02% Tween-20, 2% DMSO, and pH 7.2) and injected into ligand channel at a flow rate of 3 μl/min and a contact time of 10 min at 10 °C. Using this procedure, ∼8000 resonance units of PARP1 and ∼9000 resonance units of TNKS1 was captured on the SA surface via the biotin–streptavidin interaction. The functionalized surface was then equilibrated with assay buffer. An unfunctionalized channel was used as a reference surface for binding kinetic analysis.

#### Single-cycle kinetics

A threefold seven-point serial dilution of test compounds was set up in a deep-well 96-well microplate (Greiner; catalog no. 780201) with concentration ranging from 0 to 100 nM. Binding kinetics was measured at 25 °C in a single-cycle kinetics format by injecting serial dilution of compounds onto reference and ligand channel at a flow rate of 100 μl/min and association time of 200 s. Compound dissociation was monitored for 2000 s. Three buffer blanks were also run before each compound run for double referencing. No additional regeneration was used, and no DMSO correction was performed here. Data analysis was performed using Biacore S200 evaluation software. The double-referenced and solvent-corrected data were fit to 1:1 Langmuir model to obtain binding constant (*K*_D_) and binding kinetics (*k*_on_ and *k*_off_) information. The adequateness of the fit was judged by χ^2^ values (lower than 5% of the *R*_max_) and the randomness of residue distribution.

#### Multicycle kinetics

A twofold ten point serial dilution of test compounds was set up in a 96-well microplate (Greiner; catalog no. 650101) with concentration ranging from 0 to 100 μM. Binding kinetics was measured at 25 °C in a multicycle kinetics format by injecting serial dilution of compounds onto reference and ligand channel at a flow rate of 100 μl/min and association time of 90 s. Compound dissociation was monitored for 600 s. Two buffer blanks were also run before each compound run for double referencing. No additional regeneration was used. DMSO calibration curve was obtained before and after compound analysis by injecting 0 to 5% of DMSO in running buffer. Data analysis was performed using Biacore S200 evaluation software. The double-referenced and solvent-corrected data were fit to 1:1 Langmuir model to obtain binding constant (*K*_D_) and binding kinetics (*k*_on_ and *k*_off_) information. The adequateness of the fit was judged by χ^2^ values (lower than 5% of the *R*_max_) and the randomness of residue distribution.

### HDX–MS

TNKS1 and PARP1 samples were diluted to 10 μM with working buffer (25 mM Tris, pH = 7.2, 150 mM NaCl) prior to deuterium exchange. For protein:ligand complexes, 50 μM ligand (1% residual DMSO) was preincubated at 23 °C with protein for 1 h (Apo protein also included 1% residual DMSO as vehicle). Samples were then aliquoted (4 μl) into vials, for subsequent deuterium exchange on a temperature-controlled HDX2 autosampler (Leap Technologies). Deuterium exchange buffer (D_2_O 125 mM Tris, pD = 7.6, 150 mM NaCl) was added to each vial (1:5 v/v) to provide 83.3% D_2_O at 4 °C. Deuterium exchange was conducted across five time points (10, 60, 360, 3600, and 28,800 s) and run in replicates (triplicate/quadruplicate). After a discrete exchange time, the sample was transferred by a chilled syringe and quenched/denatured at 1 °C with addition of 40 μl of chilled quench buffer (3.2 M guanidine hydrochloride and 0.8% formic acid). Quenched samples were injected into the chiller box, which housed the sample loop, protease column, and trap/analytical columns at 3 °C. Blank injections were inserted between every sample to minimize potential carryover.

After exchange was arrested, samples were digested inline via a Vanquish UPLC pump (Dionex) running 0.1% formic acid at 200 μl/min across the pepsin/protease XIII-immobilized column (NovaBio Assay) and 2.1 × 5 mm CSH C18 trap column (Waters) for 2 min. Peptides were then separated across a Kinetex C18 2.1 × 30 mm, 1.3 micron analytical column with a 5.7-min gradient of 8 to 37% acetonitrile, 0.1% formic acid, from a Vanquish pump with mobile phase A (0.1% formic acid) and mobile phase B (100% acetonitrile and 0.1% formic acid) at 150 μl/min.

Mass spectra were acquired on a Thermo Fusion-Lumos mass spectrometer running XCalibur 2.1 with a scan range of 375 to 1300 *m*/*z*. Orbitrap resolution was set at 60,000 for charge state selection of +2 to +5 peptides. Peptide fragmentation for peptide identification employed HCD tandem MS2 acquisition. Electrospray source settings were set at high gas flow (27 sheath and nine auxillary) and temperature (150 °C) to handle the 150 μl/min LC flow rate, while minimizing potential deuterium back exchange. Peptide pools were generated in Thermo Proteome Discoverer 2.2 with a directed search against the protein sequence, using Sequest HT search and a fixed value PSM validator (0.05 Delta Cn). Tolerances were set at a mass accuracy of 5 ppm with 0.2 Da fragment. Deuterium exchange was determined with HDExaminer 3.0 software. For peptide deuterium uptake, the first two residues and prolines were excluded from calculations. HDX–MS parameters for PARP1 and TNKS1 are tabulated in Tables S2 and S3, respectively.

### Protein expression and purification

N-terminally hexahistidine-tagged TNKS1 ART domain (residues: 1104–1314) was cloned into a modified pET28a vector and was expressed in *Escherichia coli* BL21(DE3) cells grown overnight in autoinduction media at 15 °C. TNKS1 ART domain was purified using Ni^2+^ affinity chromatography followed by overnight tobacco etch virus protease incubation, followed by a subsequent Ni^2+^ affinity step, and finally gel filtration using HiPrep 26/60 Superdex-200 (GE Healthcare). Protein was concentrated to 10 to 20 mg/ml and flash frozen in storage buffer consisting of 20 mM Hepes 7.5, 300 mM NaCl, 10% glycerol, and 2 mM TCEP.

N-terminally hexahistidine-tagged full-length PARP1 and the catalytic domain (residues: 662–1011) were cloned into a modified pFastBac vector and expressed in Sf21 cells at 72 and 48 h, respectively. The catalytic domain was purified using the same purification scheme as TNKS1 ART domain. Protein was concentrated to 10 to 20 mg/ml in a storage buffer consisting of 20 mM Tris pH 7.4, 150 mM NaCl, and 1 mM TCEP.

The full-length PARP1 was purified using Ni-affinity chromatography, followed by heparin sepharose column and finally gel filtration. The His-tag was left intact at the N terminus of the protein. Protein was concentrated to 5 mg/ml in storage buffer consisting of 20 mM Hepes 7.5, 150 mM NaCl, and 1 mM TCEP and flash frozen in liquid nitrogen.

### Crystallization of TNKS1 and PARP1 proteins

Cocrystals of TNKS1 and PARP1 with the various inhibitors were grown at 4 and 21 °C, respectively, using sitting-drop vapor-diffusion method using commercial screens. Threefold molar excess of inhibitor was added to 10 to 20 mg/ml protein and incubated on ice for 60 min. Crystallization drops consisted of a 1:1 ratio of protein–inhibitor complex to well reservoir. Crystals typically grew within 1 to 7 days. PARP1 and TNKS1 crystallization conditions for PARP1 and TNKS1 are listed in Tables S4 and S5, respectively.

### X-ray data collection, structure determination, and refinement

X-ray data were collected at Industrial Macromolecular Crystallography Association Collaborative Access Team 17-ID beamline of the Advanced Photon Source. Data were processed using autoPROC (36).

PARP1 catalytic domain structure in complex with veliparib was determined using molecular replacement. The published structure (Protein Data Bank [PDB] ID: 5ws1) was used as the starting model. The structure was iteratively refined using autoBUSTER (37) and coot (38). The refined structure served as a starting model for all the other PARP1 catalytic domain structures. Where necessary, program phaser was used for molecular replacement prior to refinement.

Likewise, TNKS1 ART domain structure was determined using molecular replacement. The published structure (PDB ID: 2rf5) was used as the starting model. The structure was iteratively refined using autoBUSTER (37) and coot (38). The refined structure served as a starting model for all the other TNKS1 ART domain structures. Where necessary, program phaser was used for molecular replacement prior to refinement.

## Data availability

All structures described here (and the structure factors) have been deposited in the PDB under accession codes 7KK2, 7KK3, 7KK4, 7KK5, 7KK6, 7KKM, 7KKN, 7KKO, 7KKP, and 7KKQ. The association of the structures with the accession codes is defined in Tables 2 and 3.

## Conflict of interest

All authors are employees of Pfizer, Inc.
