# Supplementary material for: Dissecting the molecular determinants of clinical PARP1 inhibitor selectivity for tankyrase1
Source: J Biol Chem. 2021 Jan 9;296:100251. doi: 10.1074/jbc.RA120.016573 (PMC7948648; doi:10.1074/jbc.RA120.016573)
Supplement: Supplementary file 1 — Supplementary material [file mmc1.pdf]

Dissecting the molecular determinants of clinical PARP1 inhibitor selectivity for tankyrase1

Kevin Ryan[1], Ben Bolaňos[1], Marissa Smith[2], Prakash Palde[2], Paulina Delgado Cuenca[2], Todd L. VanArsdale[2], Sherry Niessen[2], Lianglin Zhang[2], Douglas Behenna[3], Martha A. Ornelas[3], Khanh T. Tran[3], Stephen Kaiser[1], Lawrence Lum[2], Al Stewart[1], Ketan S. Gajiwala[1*]

[1]Structural Biology and Protein Science, [2]Oncology Research Unit, [3]Oncology Medicinal Chemistry, Pfizer Worldwide Research and Development, Pfizer, San Diego

*Corresponding author: Ketan S. Gajiwala

E-mail: ketan.gajiwala@pfizer.com

Running Title: PARP1 inhibitor selectivity for tankyrase1

Keywords: PARP1, tankyrase1, talazoparib, olaparib, niraparib

Supporting information

Inhibitor	k_{on} ($M^{-1}. s^{-1}$)	k_{off} (s^{-1})	$t_{1/2}$ (mi n)	Kin. K_D (nM)	Fold increase in affinity[c]	Fold increase in on-rate[c]	Fold change in off-rate[c]
Talazoparib	6.0×10^5	9.2×10^{-5}	125	0.2 ± 0.01	4	5	1
Olaparib	5.2×10^5	8.9×10^{-5}	132	0.2 ± 0.02	5	5	1
Veliparib	1.2×10^6	1.8×10^{-3}	7	1.5 ± 0.2	3	2	1
Niraparib	4.9×10^5	1.3×10^{-3}	12	2.5 ± 0.8	2	1	1

[a] Binding kinetics measured in 25 mM HEPES, 500 mM NaCl, 5 % glycerol, 0.5 mM TCEP, 2 % DMSO, 0.02 % Tween 20, pH 7.4 at 25 °C. [b] Each value in the table represents an average of 2 or more independent measurements. [c] Relative to the values at 150 mM NaCl reported in Table 1.

Table S1. Kinetics of inhibitor binding to PARP1 at high salt concentration.[a] [b]

Data Set	PARP1 (control)	PARP1 catalytic domain	PARP1 + Talazoparib	PARP1 + Olaparib	PARP1 + Niraparib
HDX time course (sec)	10, 60, 360, 3600, 28800	10, 60, 360, 3600, 28800	10, 60, 360, 3600, 28800	10, 60, 360, 3600, 28800	10, 60, 360, 3600, 28800
# of peptides	446	131	445	445	443
Sequence coverage	97.64%	32.51%	97.64%	97.64%	97.64%
Average peptide length / Redundancy	16.27 / 7.15	16.27 / 2.10	16.21 / 7.11	16.27 / 7.13	16.29 / 7.11
Replicates	4	4	3	4	4
Repeatability (avg. stddev of #D)	0.1343	0.0699	0.1415	0.1032	0.132
Significant differences in HDX (delta HDX > X D - 95% CI)	n/a	0.3369 D	0.5097 D	0.3345 D	0.3614 D

Table S2: Data acquisition and analysis parameters of HDX-MS experiment on PARP1 and its complexes

Data Set	TNKS1 (control)	TNKS1 + Talazoparib	TNKS1 + Olaparib	TNKS1 + Niraparib
HDX time course (sec)	10, 60, 360, 3600, 28800	10, 60, 360, 3600, 28800	10, 60, 360, 3600, 28800	10, 60, 360, 3600, 28800
# of peptides	182	183	184	182
Sequence coverage	96.71%	96.71%	96.71%	96.71%
Average peptide length / Redundancy	15.29 / 13.07	15.36 / 13.20	15.31 / 13.23	15.37 / 13.13
Replicates	3	3	3	4
Repeatability (avg. stddev of #D)	0.1221	0.183	0.1418	0.1387
Significant differences in HDX (delta HDX > X D - 95% CI)	n/a	0.5869 D	0.4959 D	0.4855 D

Table S3: Data acquisition and analysis parameters of HDX-MS experiment on TNKS1 and its complexes

Compound (excess over [protein])	[Protein] mg/ml	Temperature °C	Conditions
Apo	25	4	0.1 M Bis Tris pH 5.5 + 25% PEG-3350 + 0.2 M LiSO4
Talazoparib (3x)	10	21	0.1 M Na-Citrate pH 5.6 + 23% PEG-4000 + 1M Ammonium Formate
Olaparib (3x)	21	21	0.1 M TRIS pH 8.5 + 0.2 M MgCl2 + 30% PEG 4000
Niraparib (3x)	10	21	0.1 M HEPES 7.5 + 25% PEG 3350 + 0.2 M Ammonium Sulfate
Veliparib (3x)	21	4	0.1 M HEPES pH 7.0 + 3 M Ammonium sulfate

Table S4: Crystallization conditions for PARP1 complexes

Compound (excess over [protein])	[Protein] mg/ml	Temperature °C	Conditions
Apo	10.5	4	0.1 M MES pH 6.0 + 0.1M NaCl + 10% PEG 4000 + 20% Ethylene glycol
Talazoparib (2.5x)	10.5	4	0.1 M Sodium acetate pH 5.0 + 2.3 M Ammonium formate
Olaparib (2.5x)	10.5	4	0.1 M MES pH 6.14 + 0.4 M Potassium acetate + 22% PEG 4000
Niraparib (2.5x)	10.5	4	0.1 M Sodium acetate pH 5.5 + 0.2 M Lithium sulfate + 10% PEG 1500 + 30% IPA
Veliparib (2.5 x)	10.9	4	0.1 M Sodium citrate pH 5.6 + 1 M Ammonium formate + 23 % PEG 4000

Table S5: Crystallization conditions for TNKS1 complexes

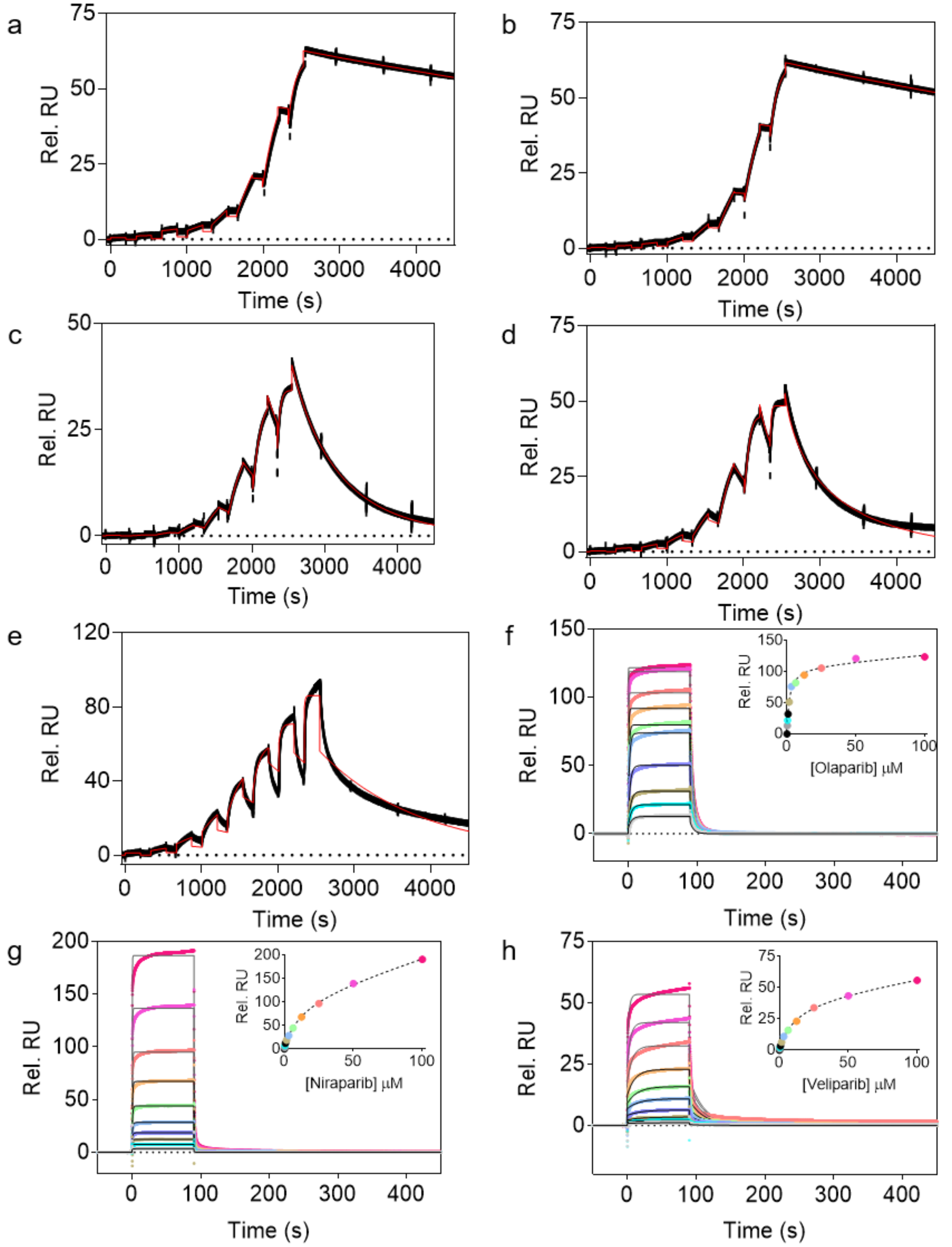

Figure S1. Measurement of PARP1 (**a - d**) and TNKS1 (**e - h**) binding kinetics using SPR with respect to talazoparib, olaparib, niraparib and veliparib, respectively.

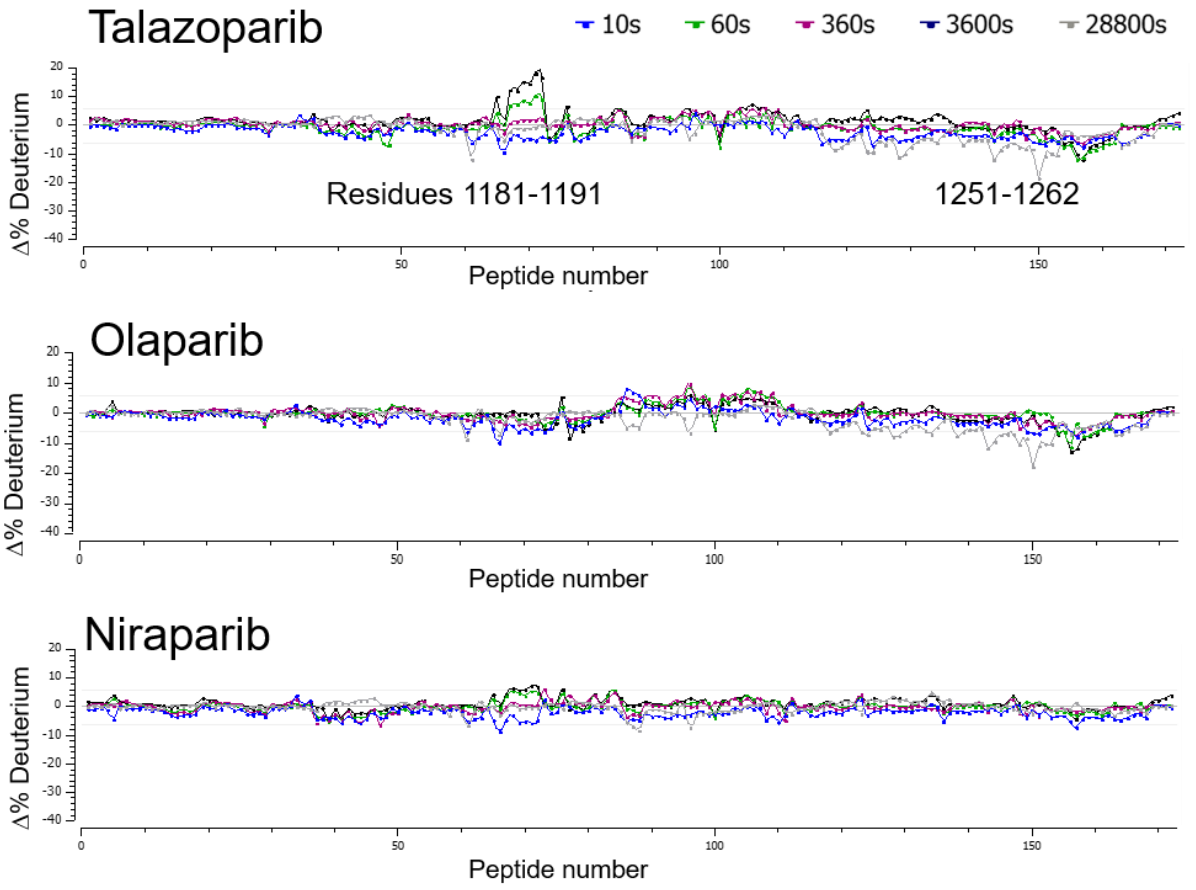

Figure S2. Differential HDX profiles of TNKS1. The deuterium uptake of the TNKS1-ART domain in the presence of the indicated ligand is shown relative to that of the apo-protein.

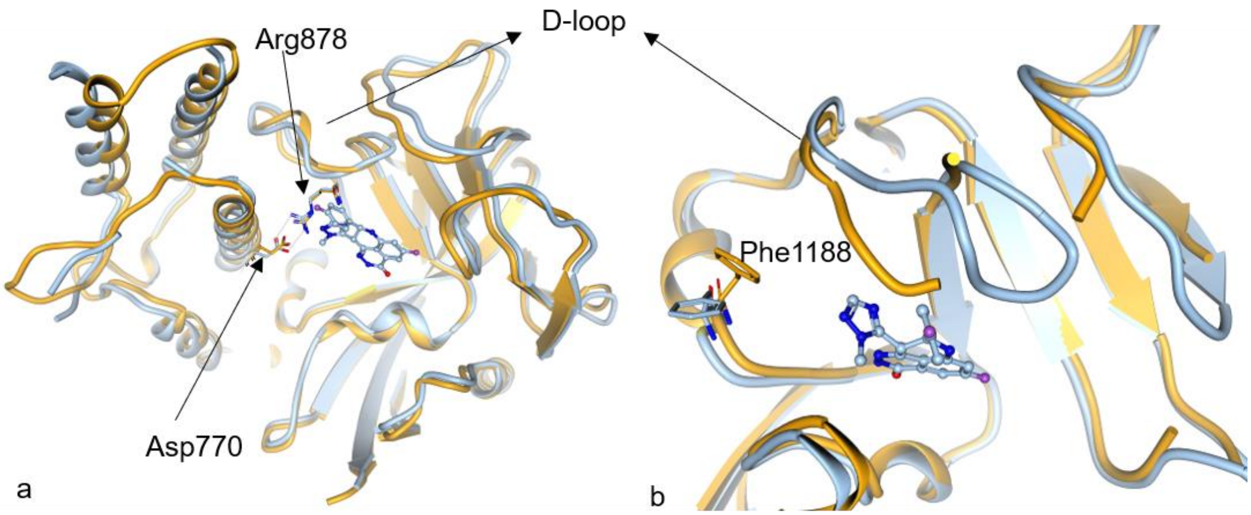

Figure S3. Comparison of the apo-protein (orange) with talazoparib-bound protein (blue). (a) PARP1 structure in the presence and absence of the inhibitor shows the same local conformation with some rigid body motions. In both forms, Arg878 from the D-loop ion pairs with Asp770 from the helical domain, likely constraining its conformation. (b) TNKS1 undergoes conformational changes in the D-loop and Phe1188 orientation.

```
        8888888999 9
        6668899000 9
        2348968347 8
PARP1   HGSGYYAKSY-E
TNKS1   HGSGGYAKSYIE
        111111111111
        111222222222
        888001122229
        456563501481
```

Figure S4. The structure guided sequence alignment of the residues within 4 Å of the talazoparib binding site of PARP1 and TNKS1. Ile1228 in TNKS1 belongs to a loop that is flexible by virtue of the presence of multiple Gly residues. It does not seem to make consistent contact with the inhibitor molecules and may contribute little to the ligand recognition.

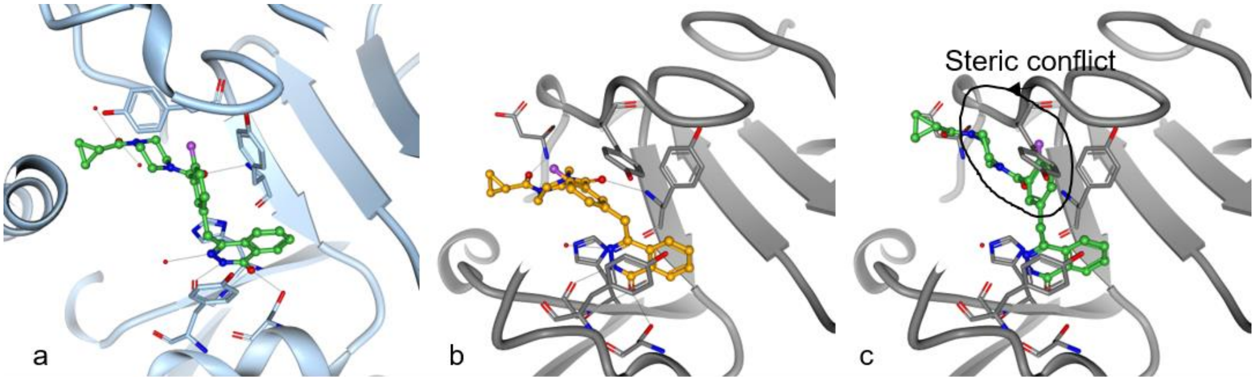

Figure S5. PARP1 and TNKS1 recognition of olaparib. (a) PARP1 and (b) TNKS1 in complex with olaparib. (c) Modeling of PARP1-bound olaparib conformation in TNKS1 structure results in severe steric conflicts with the D-loop.

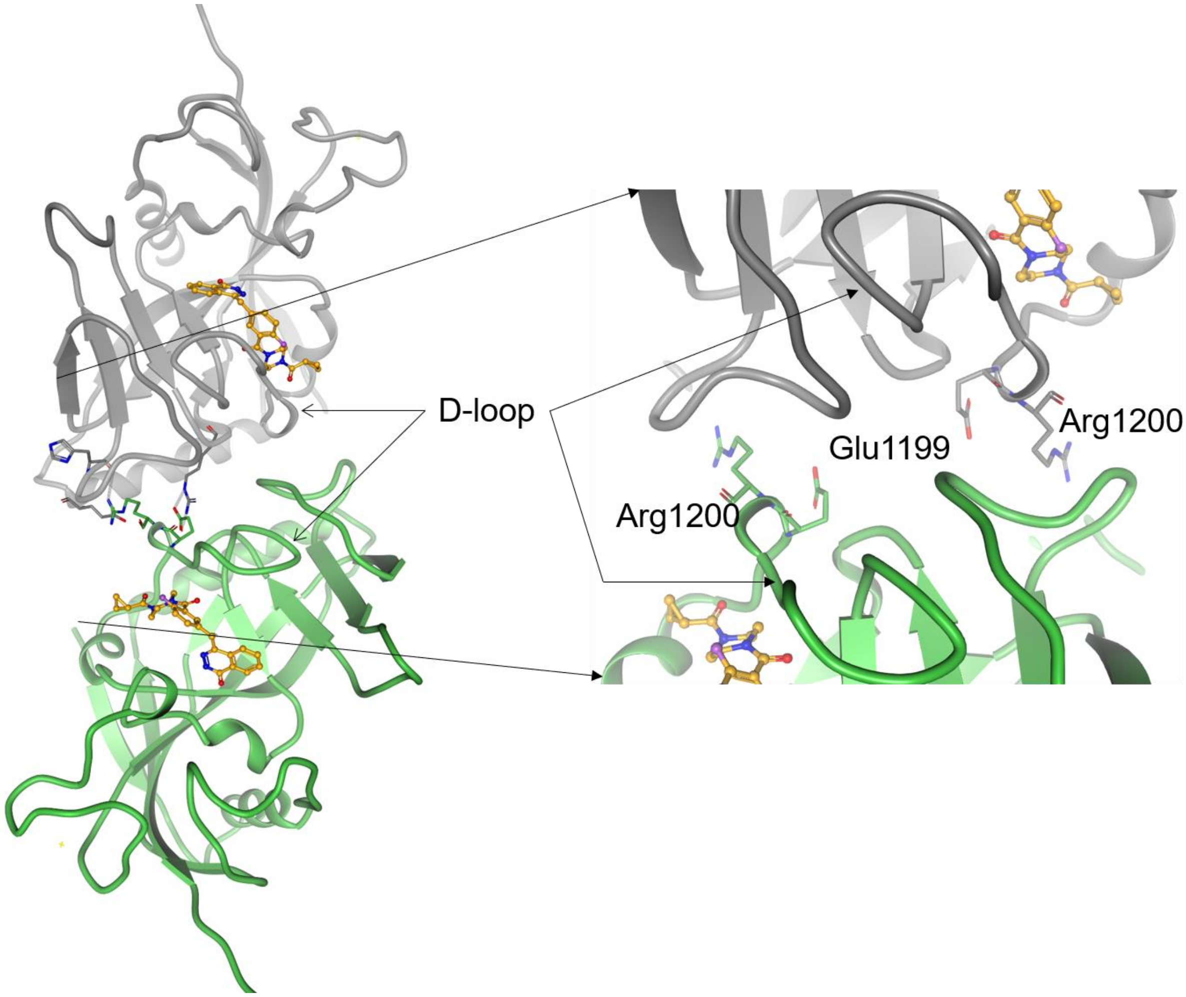

Figure S6. Proposed functional dimer of TNKS1, where Glu1199 and Arg1200 from the D-loop are engaged in dimeric interactions. For clarity only one set of interactions are shown in the left panel. Each TNKS1 molecule is shown in a different color. Bound olaparib is shown in orange.

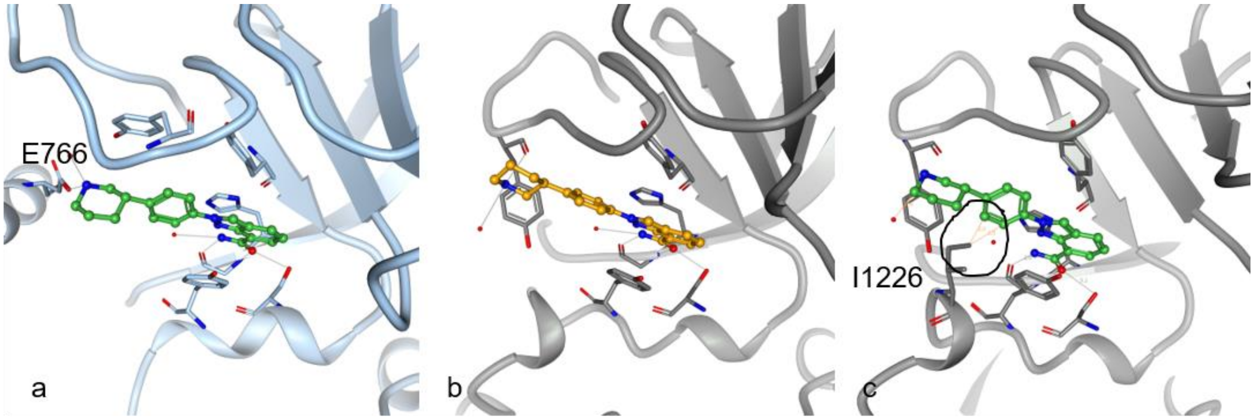

Figure S7. Niraparib recognition by (a) PARP1 and (b) TNKS1. TNKS1 with lowered D-loop and fully modeled ligand is shown. (c) Modeling of PARP1-bound niraparib conformation into TNKS1 structure shows steric conflict between Ile1228 and the ligand, highlighted by a circle.

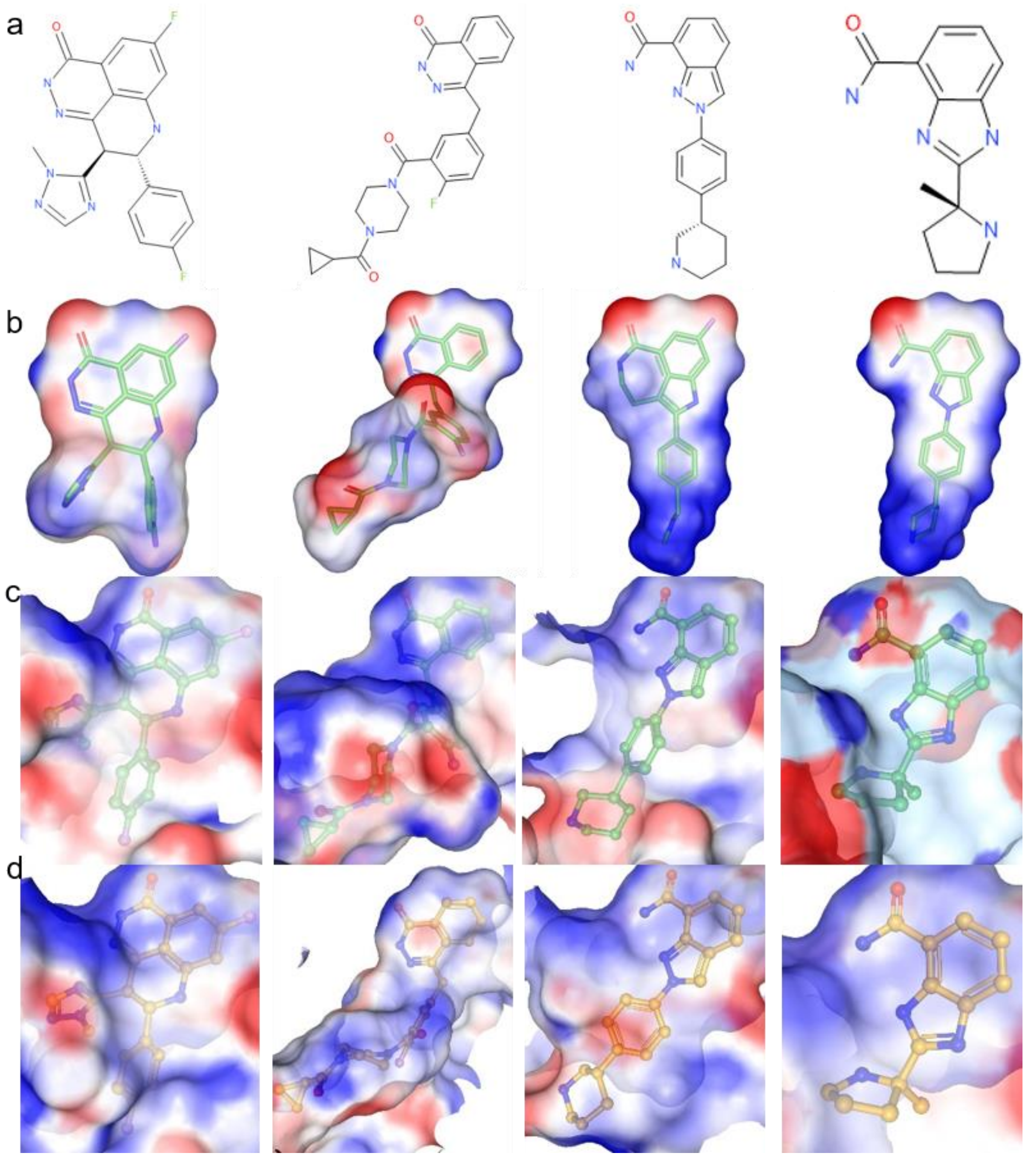

Figure S8. Electrostatics of the ligands and the ligand binding sites of PARP1 and TNKS1. Row a: chemical structures of the inhibitors; row b: the electrostatic surface representation of the inhibitors; rows c and d: electrostatic surface representation of the ligand bindings sites of PARP1 and TNKS1, respectively. Negative surface potential is shown in red and positive in blue. From left to right: talazoparib, olaparib, niraparib and veliparib.

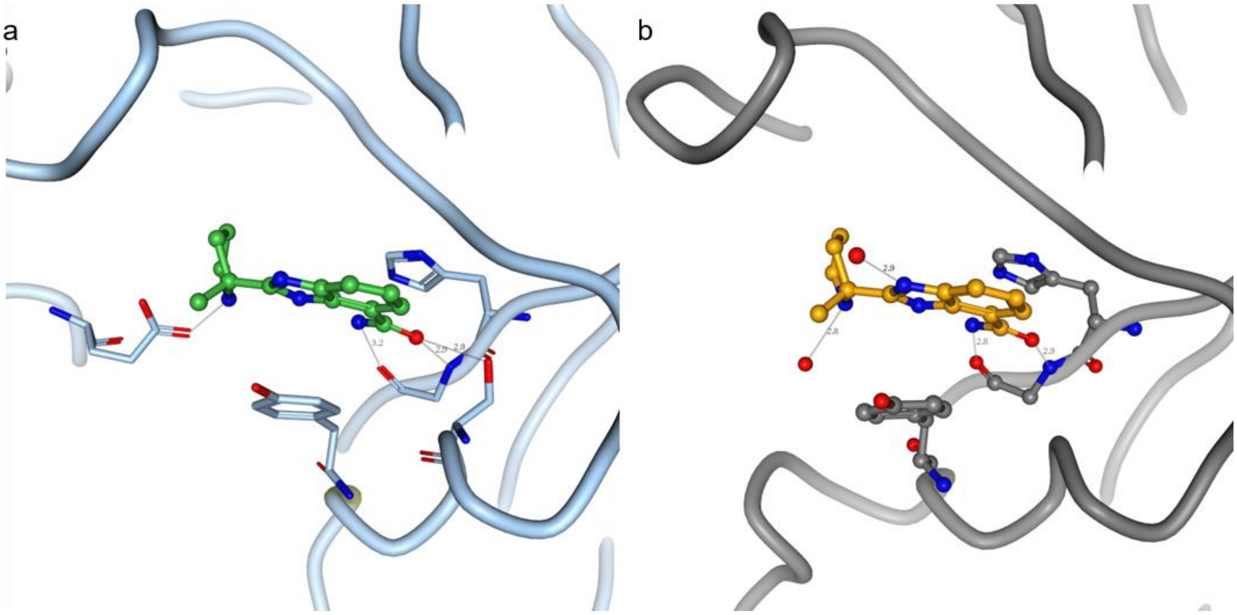

Figure S9. Veliparib recognition by (a) PARP1 and (b) TNKS1.

Synthetic Procedures

Starting materials and reagents were purchased from commercial suppliers and were used without further purification unless otherwise indicated. Reactions were performed under a nitrogen atmosphere, were assayed by high-performance liquid chromatography (HPLC) or thin-layer chromatography (TLC), and terminated as judged by the consumption of starting material. Analytical thin-layer chromatography was performed on glass-backed silica gel 60_F 254 plates (Analtech (0.25 mm)) and eluted with the appropriate solvent ratios (v/v). TLC plates were visualized by UV fluorescence, phosphomolybdic acid stain, anisaldehyde stain or iodine stain. ^{1}H NMR spectra were recorded on a Bruker instrument operating at 400 MHz unless otherwise indicated. ^{1}H NMR spectra were obtained as DMSO-d_6 or $CDCl_3$ solutions as indicated (reported in parts per million) using $CDCl_3$ as the reference standard (7.27 ppm) or DMSO-d_6 (2.50 ppm) at 30 °C unless otherwise noted. ^{13}C NMR spectra were referenced internally to solvent. ^{19}F spectra were referenced using the unified scale method with the field locked on an internal deuterium resonance[1], and are broadband decoupled unless otherwise noted. When peak multiplicities are reported, the following abbreviations are used: s = singlet, d = doublet, t = triplet, q = quartet, quin. = quintet, m = multiplet, br. = broad, dd = doublet of doublets, dt = doublet of triplets. Coupling constants, when given, are reported in hertz. Mass spectra were obtained using liquid chromatography−mass spectrometry (LCMS) on an Agilent 1260 LC with MSD Agilent model 6120 single-quadrupole mass spec detectors using atmospheric pressure chemical ionization (APCI) or electrospray ionization (ESI). High-resolution mass measurements were carried out on an Agilent TOF 6200 series using ESI methods. All test compounds showed >95% purity as determined by HPLC. Analytical HPLC conditions were as follows: WatersAcquityBEH C18, 2.1x30mm, 1.7µm particle size, 5-95 %B in 2.5 min, 95%B 2.5-3.0 min; Flow rate 1.2 mL/min (Solvent A: Water (0.1% formic acid + 0.05% ammonium formate) and Solvent B: Acetonitrile (5% H_2O + 0.1% formic acid + 0.05% ammonium formate)); UV detection (λ = 254, 220 nm).

Synthetic Schemes

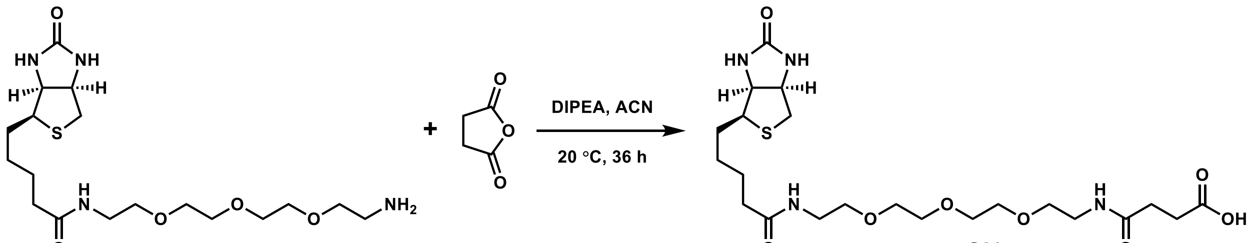

[1] For a description of the method, see: Harris, R. K.; Becker, E. D.; Cabral De Menezes, S. M.; Granger, P.; Hoffman, R. E.; Zilm, K. W. *Pure Appl. Chem.*, **2008**, *80* (1), 59-84.

Scheme 1

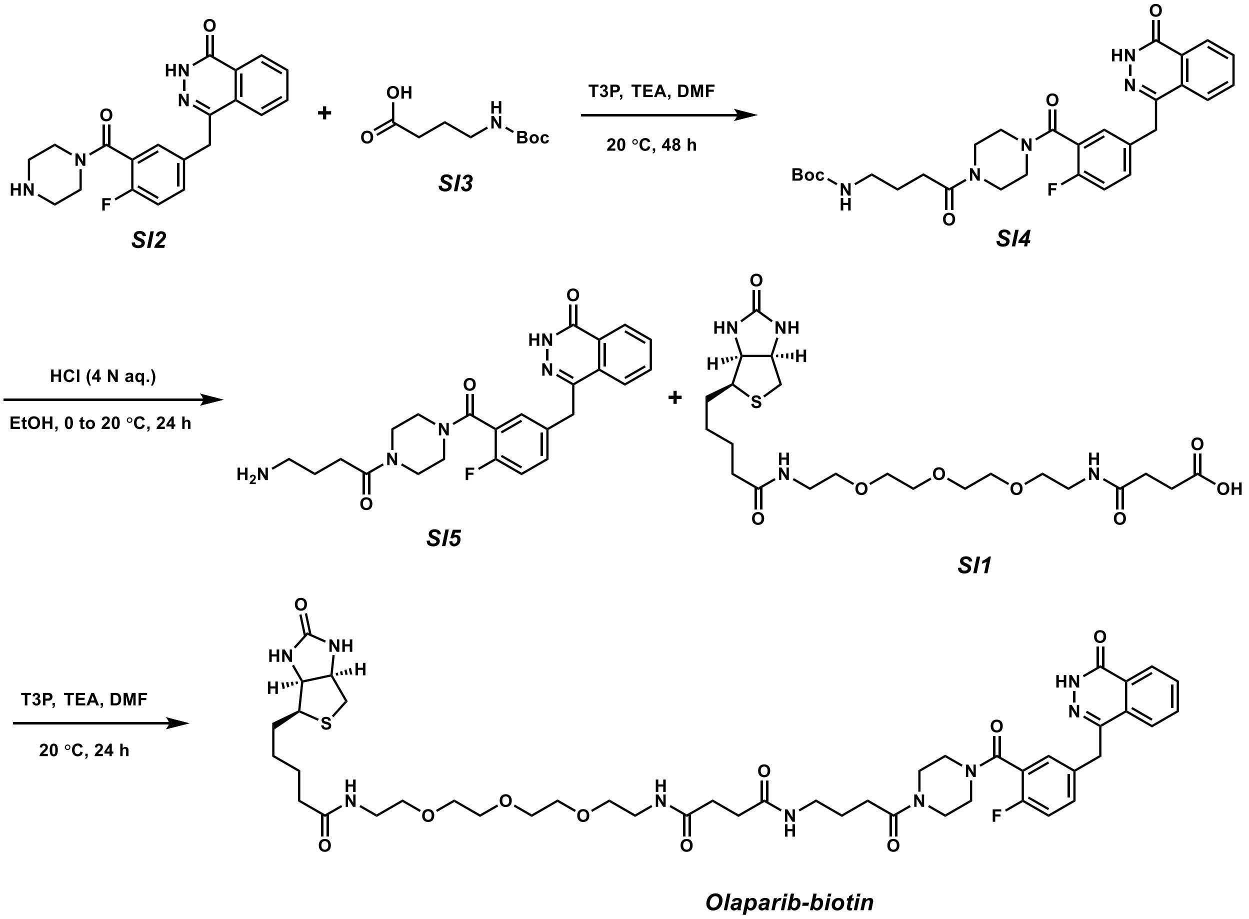

Scheme 2

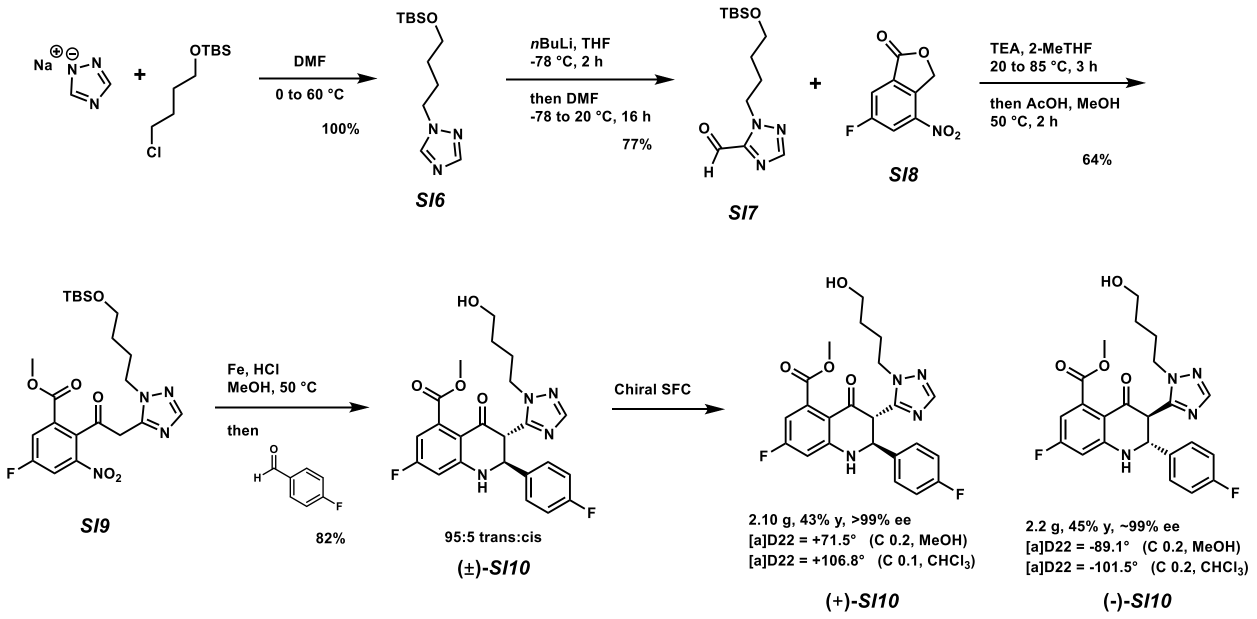

DMF
0 to 60 °C
100%
nBuLi, THF
-78 °C, 2 h
then DMF
-78 to 20 °C, 16 h
77%
TEA, 2-MeTHF
20 to 85 °C, 3 h
then AcOH, MeOH
50 °C, 2 h
64%
SI6
SI7
SI8
SI9
Fe, HCl
MeOH, 50 °C
then
82%
Chiral SFC
95:5 trans:cis
(±)-SI10
2.10 g, 43% y, >99% ee
[a]D22 = +71.5° (C 0.2, MeOH)
[a]D22 = +106.8° (C 0.1, CHCl3)
(+)-SI10
2.2 g, 45% y, ~99% ee
[a]D22 = -89.1° (C 0.2, MeOH)
[a]D22 = -101.5° (C 0.2, CHCl3)
(-)-SI10

Scheme 3

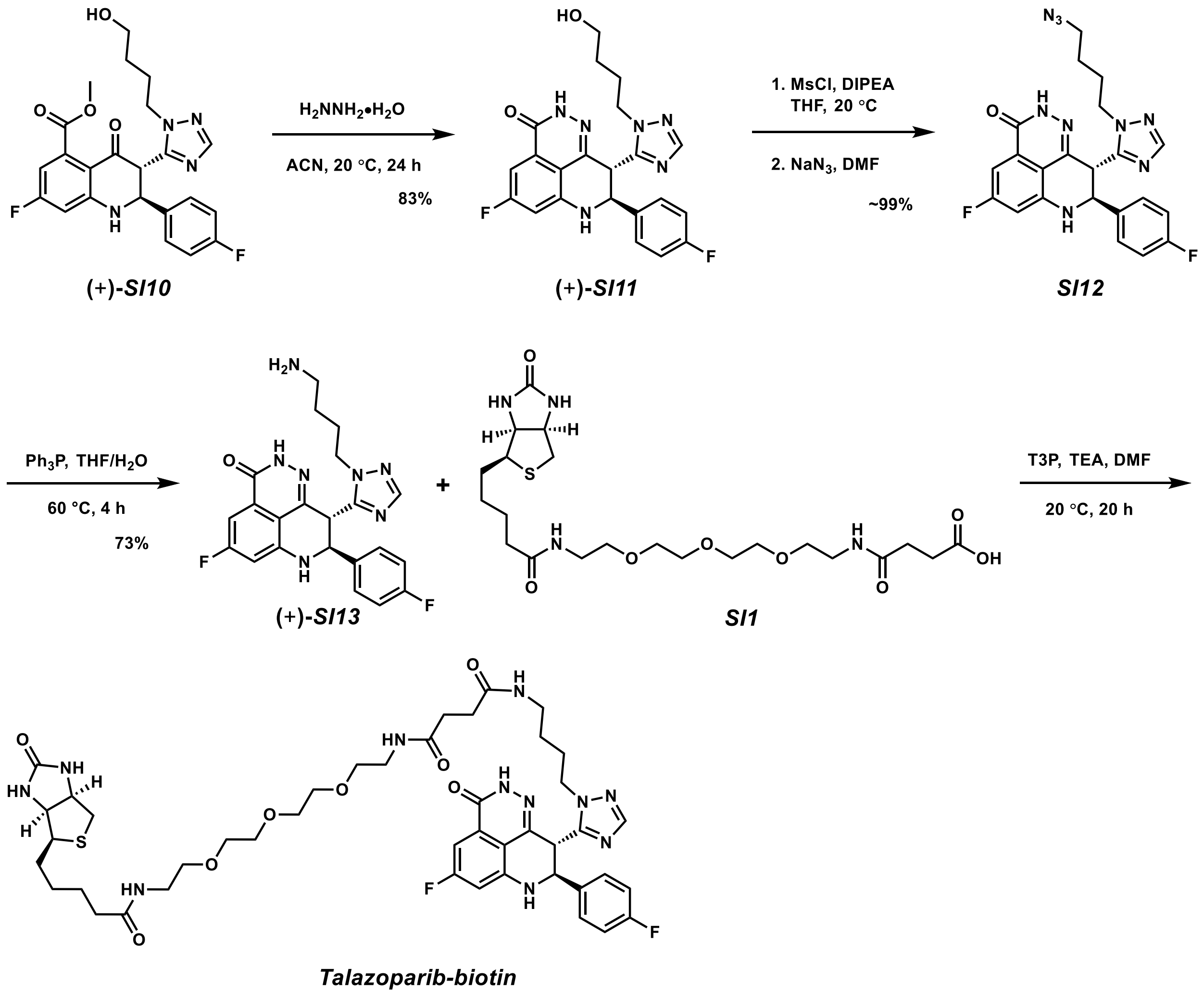

Scheme 4

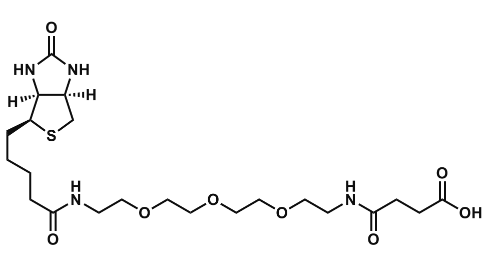

4,18-dioxo-22-[(3a*S*,4*S*,6a*R*)-2-oxohexahydro-1*H*-thieno[3,4-*d*]imidazol-4-yl]-8,11,14-trioxa-5,17-diazadocosan-1-oic acid (SI1).[2] A vial was charged with *N*-(2-{2-[2-(2-

---

[2] For previous syntheses, see (a) Han, B.; Stevens, J. F.; Maier, C. S. *Anal. Chem.* **2007**, *79* (9), 3342-3354; (b) Trester-Zedlitz, M.; Kamada, K.; Burley, S. K.; Chait, B. T.; Muir, T. W. *J. Am. Chem. Soc.* **2003**, *125* (9), 2416-2425.

aminoethoxy)ethoxy]ethoxy}ethyl)-5-[(3a*S*,4*S*,6a*R*)-2-oxohexahydro-1*H*-thieno[3,4-*d*]imidazol-4-yl]pentanamide (1.10 g, 2.63 mmol, 1.00 equiv.), acetonitrile (5 ml, 0.5 M), DIPEA (2.18 ml, 13.1 mmol, 5.00 equiv.) and succinyl anhydride (526 mg, 5.26 mmol, 2.00 equiv.). The resulting suspension was stirred at ambient temperature for 36 h, diluted with MeOH (10 ml) and purified by RP-HPLC to give the title compound as a white powder after lyophilization (1.02 g, 75%). ^{1}H NMR (400MHz, DMSO-d$_6$) δ 12.05 (br. s, 1H), 7.89 (t, *J*=5.5 Hz, 1H), 7.82 (t, *J*=5.6 Hz, 1H), 6.41 (s, 1H), 6.35 (s, 1H), 4.30 (dd, *J*=5.3, 7.6 Hz, 1H), 4.16 - 4.07 (m, 1H), 3.55-3.45 (m, 8H), 3.41-3.35 (m, 4H), 3.32 (s, 1H), 3.18 (q, *J*=5.8 Hz, 4H), 3.13 - 3.06 (m, 1H), 2.82 (dd, *J*=5.1, 12.4 Hz, 1H), 2.57 (d, *J*=12.3 Hz, 1H), 2.44 - 2.26 (m, 3H), 2.06 (t, *J*=7.5 Hz, 2H), 1.67 - 1.55 (m, 1H), 1.55 - 1.39 (m, 3H), 1.37 - 1.21 (m, 2H).

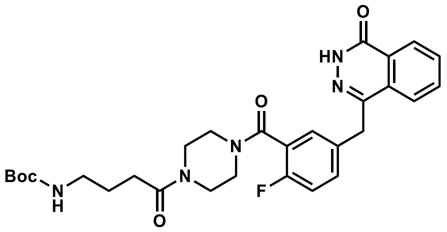

***tert*-butyl (4-(4-(2-fluoro-5-((4-oxo-3,4-dihydrophthalazin-1-yl)methyl)benzoyl)piperazin-1-yl)-4-oxobutyl)carbamate (SI4).**[3] A vial was charged with 4-(4-fluoro-3-(piperazine-1-carbonyl)benzyl)phthalazin-1(2*H*)-one (1.00 g, 2.73 mmol, 1.00 equiv.), 4-((*tert*-butoxycarbonyl)amino)butanoic acid (693 mg, 3.41 mmol, 1.25 equiv.), TEA (1.15 ml, 8.19 mmol, 3.00 equiv.) and DMF (9.1 ml, 0.3 M). To this suspension was added T3P (50% in EtOAc, 2.44 ml, 4.09 mmol, 1.50 equiv.), the resulting homogenous solution produced a slight exotherm (to *ca.* 35 °C, and was stirred at ambient temperature for 48 h. The reaction mixture was quenched into water (125 ml) and EtOAc (50 ml). The aqueous layer was extracted (2 x 25 ml EtOAc), and the combined aqueous layers were washed with brine, dried (Na_2SO_4), and concentrated to give the title compound as a solid, which was used without further purification (1.75 g, ~100%, heavy with DMF). ^{1}H NMR (400 MHz, DMSO-d$_6$) δ 12.60 (s, 1H), 8.26 (d, *J*=7.7 Hz, 1H), 7.97 (s, 1H), 7.89 (t, *J*=7.5 Hz, 1H), 7.85 - 7.77 (m, 1H), 7.44 (br. s, 1H), 7.36 (br. s, 1H), 7.23 (t, *J*=9.0 Hz, 1H), 6.79 (d, *J*=5.4 Hz, 1H), 4.33 (s, 2H), 3.68 - 3.55 (m, 2H), 3.51 (br. s, 2H), 3.36 (d, *J*=15.0 Hz, 2H), 3.17 (d, *J*=15.4 Hz, 2H), 2.98 - 2.90 (m, 2H), 2.37 - 2.23 (m, 2H), 1.60 (d, *J*=6.5 Hz, 2H), 1.40 - 1.31 (m, 9H); LRMS (APCI+) m/z calc'd for $C_{29}H_{35}FN_5O_5$ $[M+H]^+$ 552.3, found 552.1.

[3] For previous synthesis of this compound, see: Knezevic, C. E.; Wright, G.; Remsing Rix, L. L.; Kim, W.; Kuenzi, B. M.; Luo, Y.; Watters, J. M.; Koomen, J. M.; Haura, E. B.; Monteiro, A. N.; Radu, C.; Lawrence, H. R. *Cell Chem. Bio.* **2016**, *23*, 1490-1503.

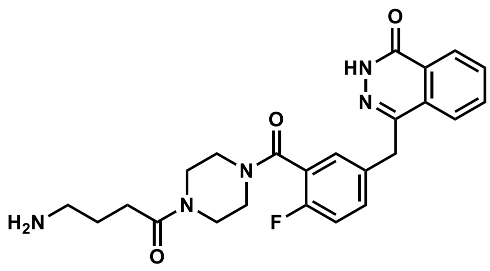

4-(3-(4-(4-aminobutanoyl)piperazine-1-carbonyl)-4-fluorobenzyl)phthalazin-1(2*H*)-one (SI5). To a cooled (0 °C) solution of crude **SI4** (~2.73 mmol) in EtOH (12 ml) was added aqueous HCl (4 N, 12 ml). The reaction mixture was allowed to warm to ambient temperature overnight, and after 24 h the reaction was quenched with sat. aq. $NaHCO_3$ (100 ml), and the EtOH was removed under reduced pressure. The aqueous layer was washed with DCM (5 x 25 ml), and then concentrated to dryness. The residue was suspended in sat. aq. $NaHCO_3$ (50 ml) and extracted with 3/1 $CHCl_3$/*i*PrOH (6 x 40 ml). The aqueous layer was found to contain significant product and was concentrated to dryness. The residue was washed with 1/4/4 DMSO/DCM/EtOH (100 ml). The combined organics were concentrated under reduced pressure to give a DMSO solution that was purified by SFC on a ZymorSPHER 4-Pyridine column using MeOH containing 10 mM NH_3 to give the title compound as a solid (507 mg, 41%). ^{1}H NMR (400 MHz, DMSO-d_6) δ 12.61 (br. s, 1H), 8.26 (d, *J*=7.7 Hz, 1H), 8.00 - 7.94 (m, 1H), 7.89 (t, *J*=7.4 Hz, 1H), 7.86 - 7.80 (m, 1H), 7.45 (br. s, 1H), 7.36 (t, *J*=6.4 Hz, 1H), 7.24 (t, *J*=9.0 Hz, 1H), 6.36 (br. s, 1H), 4.33 (s, 2H), 3.71 - 3.46 (m, 4H), 3.32 (br. s, 2H), 3.23 - 3.08 (m, 2H), 2.77 - 2.64 (m, 2H), 2.47 - 2.34 (m, 2H), 1.70 (br. s, 2H); ^{19}F NMR (376 MHz, DMSO-d_6) δ -119.74 (s, 1F); LRMS (APCI+) m/z calc'd for $C_{24}H_{27}FN_5O_3$ $[M+H]^+$ 452.2, found 452.1.

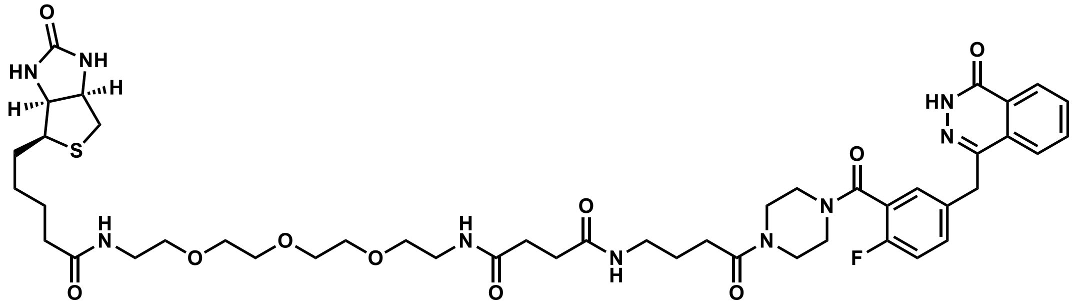

***N*¹-(4-(4-(2-fluoro-5-((4-oxo-3,4-dihydrophthalazin-1-yl)methyl)benzoyl)piperazin-1-yl)-4-oxobutyl)-*N*⁴-(13-oxo-17-((3a*S*,4*S*,6a*R*)-2-oxohexahydro-1*H*-thieno[3,4-*d*]imidazol-4-yl)-3,6,9-trioxa-12-azaheptadecyl)succinimide (olaparib-biotin).** To a solution of amine **SI5** (74.0 mg, 0.164 mmol, 1.00 equiv.), acid **SI1** (128 mg, 0.246 mmol, 1.50 equiv.), DIPEA (114 μl, 0.656 mmol, 4.00 equiv.) and DMF (2.7 ml, 0.06 M) was added T3P (50% in EtOAc, 191 μl, 0.328 mmol, 2.00 equiv.) in a dropwise manner. After 24 h, the reaction mixture was quenched with DMSO (2.5 ml) and the resulting solution was purified sequentially by RP-HPLC and then by SFC using a Princeton HA-Morpholine column (MeOH) to give the title compound as a white solid (44.2 mg, 28%). ^{1}H NMR (600 MHz, DMSO-d_6) δ 12.58 (s, 1H), 8.26 (br. d, *J*=7.7 Hz, 1H), 7.96 (d, *J*=8.1 Hz, 1H), 7.89 (br. t, *J*=7.5 Hz, 1H), 7.87 - 7.81 (m, 2H), 7.81 - 7.73 (m, 2H), 7.44 (br. s, 1H), 7.36 (br. s, 1H), 7.23 (t, *J*=8.9 Hz, 1H), 6.39 (s, 1H), 6.33 (s, 1H), 4.33 (s, 2H), 4.32 - 4.27 (m, 1H), 4.15 - 4.08 (m, 1H), 3.64 (br. s, 1H), 3.57 (br. s, 1H), 3.50 (br. s, 10H), 3.43

- 3.37 (m, 4H), 3.35 (br. s, 2H), 3.24 - 3.12 (m, 6H), 3.11 - 3.07 (m, 1H), 3.04 (br. d, *J*=5.1 Hz, 2H), 2.81 (dd, *J*=5.1, 12.5 Hz, 1H), 2.58 (d, *J*=12.3 Hz, 1H), 2.38 - 2.22 (m, 6H), 2.06 (t, *J*=7.4 Hz, 2H), 1.68 - 1.56 (m, 3H), 1.53 - 1.39 (m, 3H), 1.36 - 1.24 (m, 2H); ^{19}F NMR (565 MHz, DMSO-d_6) δ -119.7 (s, 1F); ^{13}C NMR (151 MHz, DMSO-d_6) δ 172.1, 171.5, 171.2, 170.6 (br. s, 1C), 164.0 (br. d, $J_{C\text{-}F}$=8.8 Hz, 1C), 162.7, 159.4, 156.3 (br. d, $J_{C\text{-}F}$ = 244.4 Hz, 1C), 144.8, 134.8 (br. s, 1C), 133.5 (br. s, 1C), 131.7 (br. d, $J_{C\text{-}F}$=7.7 Hz, 1C), 131.5, 129.1, 128. 9 (d, $J_{C\text{-}F}$=3.3 Hz, 1C), 127.9, 126.1, 125.4, 123.6 (br. d, $J_{C\text{-}F}$=18.8 Hz, 1C), 115.9 (br. d, $J_{C\text{-}F}$=21.0 Hz, 1C), 69.7, 69.5, 69.1, 69.1 (br. s, 1C), 61.0, 59.2, 55.4, 46.6, 46.3 (br. s, 1C), 44.9 (br. s, 1C), 44.4 (br. s, 1C), 41.6 (br. s, 1C), 41.1, 40.6 (br. s, 1C), 38.5 (br. s, 1C), 38.4, 38.1, 36.4, 35.1, 30.7 (br. s, 1C), 30.7 (br. s, 1C), 29.6, 28.2, 28.0, 25.2, 24.8; HRMS (ESI+) m/z calc'd for $C_{46}H_{62}FN_9O_{10}S$ $[M+H]^+$ 952.4397, found 952.4421.

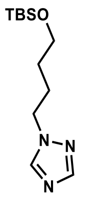

1-(4-((*tert*-butyldimethylsilyl)oxy)butyl)-1*H*-1,2,4-triazole (SI6). To a cooled (0 °C) N_2 flushed flask was added sodium triazole (6.75 g, 74.2 mmol, 1.00 equiv.) and DMF (74 ml, 1 M). To this suspension was added *tert*-butyl(4-chlorobutoxy) dimethylsilane (20.0 ml, 74.0 mmol, 1.00 equiv.) in a dropwise manner over 5 min followed by DMF (5 ml). The reaction mixture was warmed to ambient temperature for 15 min and then 60 °C for 2 h. The reaction mixture was concentrated (~5 mmHg, 30 °C), diluted with EtOAc (200 ml) and water (30 ml). The aqueous layer was extracted with EtOAc (3 x 150 ml). The combined organic layers were washed with water (2 x 30 ml), then brine (30 ml), dried (Na_2SO_4), and concentrated to give the title compound as a colorless oil (19.6 g, 100% y, ~85% purity). ^{1}H NMR (400 MHz, $CDCl_3$) δ 8.06 (s, 1H), 7.94 (s, 1H), 4.22 (t, *J*=7.2 Hz, 2H), 3.65 (t, *J*=6.1 Hz, 2H), 2.07 - 1.92 (m, 2H), 1.57 - 1.46 (m, 2H), 0.89 (s, 9H), 0.05 (s, 6H); LRMS (APCI+) m/z calc'd for $C_{12}H_{26}N_3OSi$ $[M+H]^+$ 256.2, found 256.3.

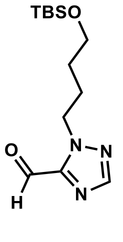

1-(4-((*tert*-butyldimethylsilyl)oxy)butyl)-1*H*-1,2,4-triazole-5-carbaldehyde (SI7). A cooled (-78 °C) N_2 flushed flask containing with triazole **SI6** (19.0 g, 85% purity, 63.0 mmol, 1.00 equiv.) and THF (126 ml, 0.5 M) was treated with *n*-BuLi (2.5 M in hexanes, 25.3 ml, 63.2 mmol, 1.00 equiv.) in a dropwise manner to maintain an internal temperature of less than -65 °C. The reaction mixture was stirred for 2 h, and DMF (4.90 ml, 63.2 mmol, 1.00 equiv.) was added in a dropwise manner over ~3 min, and then the reaction mixture was allowed to warm to ambient temperature over 16 h. The reaction mixture was cooled (0 °C), quenched with sat. aq. NH_4Cl

(30 ml), and diluted with EtOAc (300 ml). The aqueous layer was extracted with additional EtOAc (2 x 150 ml). The combined organic layers were washed with brine (20 ml), dried (Na_2SO_4), and concentrated to give a yellow oil, which was purified on silica gel (Hept/EtOAc) to give the title compound as a colorless oil (13.9 g 77%). ^{1}H NMR (400 MHz, $CDCl_3$) δ 10.01 (s, 1H), 8.03 (s, 1H), 4.61 (t, *J*=7.3 Hz, 2H), 3.63 (t, *J*=6.2 Hz, 2H), 2.00 - 1.88 (m, 2H), 1.57 - 1.48 (m, 2H), 0.88 (s, 9H), 0.04 (s, 6H); LRMS (APCI+) m/z calc'd for $C_{12}H_{26}N_3OSi$ [M-CHO+H]$^+$ 256.2, found 256.3.

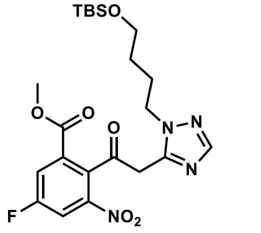

Methyl 2-(2-(1-(4-((*tert*-butyldimethylsilyl)oxy)butyl)-1*H*-1,2,4-triazol-5-yl)acetyl)-5-fluoro-3-nitrobenzoate (SI9). To a N_2 flushed flask charged with 6-fluoro-4-nitroisobenzofuran-1(3*H*)-one (**SI8**, 4.10 g, 20.8 mmol, 1.00 equiv.) and a solution of aldehyde **SI7** (7.10 g, 25.0 mmol, 1.20 equiv.) in 2-MeTHF (42 ml, 0.5 M) was added TEA (3.48 ml, 25.0 mmol, 1.20 equiv.) in a dropwise manner over ~3 min. After 10 min, the reaction mixture was heated to 85 °C under a reflux condenser for 3 h, then cooled, and concentrated under reduced pressure. The resulting red residue was diluted with MeOH (104 ml) and acetic acid (298 μL, 5.20 mmol, 0.25 equiv.) and heated at 50 °C under a reflux condenser for 2 h. The reaction mixture was concentrated and purified on silica gel (Hept/EtOAc) to give the title compound as a colorless solid (6.57 g, 64%). ^{1}H NMR (400 MHz, DMSO-d_6) δ 8.53 (dd, *J*=2.6, 8.3 Hz, 1H), 8.27 (dd, *J*=2.6, 8.3 Hz, 1H), 7.88 (s, 1H), 4.59 (s, 2H), 4.20 (t, *J*=7.4 Hz, 2H), 3.91 (s, 3H), 3.61 (t, *J*=6.3 Hz, 2H), 1.90 - 1.78 (m, 2H), 1.57 - 1.44 (m, 2H), 0.85 (s, 9H), 0.02 (s, 6H); ^{19}F NMR (376 MHz, DMSO-d_6) δ -106.9 (s, 1F); LRMS (APCI+) m/z calc'd for $C_{22}H_{32}FN_4O_6Si$ [M+H]$^+$ 495.2, found 495.3.

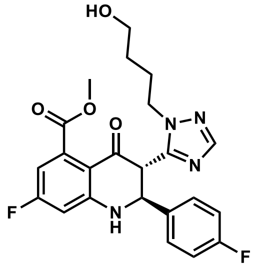

(+)-Methyl (2*S*,3*S*)-7-fluoro-2-(4-fluorophenyl)-3-[1-(4-hydroxybutyl)-1*H*-1,2,4-triazol-5-yl]-4-oxo-1,2,3,4-tetrahydroquinoline-5-carboxylate ((+)-SI10). To a cooled (0 °C) N_2 flushed flask containing MeOH (75 ml) was added acetyl chloride (8.05 ml, 113 mmol, 6.00 equiv.). After 15 min, a solution of **SI9** (7.00 g, 14.2 mmol, 1.00 equiv.) in MeOH (20 ml) was added and the reaction mixture was brought to ambient temperature. Iron powder (325 mesh, 4.74 g, 84.9 mmol, 6.00 equiv.) was added over about 3 min (slight exotherm) and stirred for an additional 15

min. The reaction mixture was filtered and the solids were discarded. To the filtrate was added 4-fluorobenzaldehyde (3.04 ml, 28.3 mmol, 2.00 equiv.) and the reaction mixture was heated at 50 °C for 40 min. The reaction mixture was then concentrated (~10 mmHg, 30 °C) to remove about one third of the methanol, and diluted with ethyl acetate (300 ml). The combined organics were washed with saturated aqueous $NaHCO_3$ (150 ml) and the layers were separated. The aqueous layer was diluted with water (100 ml) and extracted with EtOAc (2 x 250 ml). The combined organic layers were washed with 10% wt/v $Na_2S_2O_3$ (100 ml), dried (Na_2SO_4), and evaporated to give a residue, which was purified on silica gel (Hept/EtOAc) to give the racemic title compound (5.27 g, 82%) as a colorless oil containing about 5% of the cis isomer. This material was separated by chiral SFC (Chiralpak IC-3 4.6 x 100mm 3u column, 20% MeOH in CO_2 @ 120 bar, 4 mL/min. Peak 1 @ 0.77 min, Peak 2 @ 1.09 min) to give **(+)-SI10** (2.10 g, 43%, peak 1). ^{1}H NMR (400 MHz, DMSO-d_6) δ 7.87 (s, 1H), 7.82 (s, 1H), 7.53 - 7.46 (m, 2H), 7.18 - 7.09 (m, 2H), 6.74 (dd, *J*=2.4, 10.9 Hz, 1H), 6.56 (dd, *J*=2.4, 8.4 Hz, 1H), 5.30 - 4.92 (m, 2H), 4.37 (t, *J*=5.1 Hz, 1H), 3.98 - 3.84 (m, 2H), 3.72 (s, 3H), 3.30 - 3.23 (m, 2H), 1.56 - 1.41 (m, 1H), 1.40 - 1.27 (m, 1H), 1.25 - 1.04 (m, 2H); ^{19}F NMR (376 MHz, DMSO-d_6) δ -102.8 (s, 1F), -113.6 (s, 1F); LRMS (APCI+) m/z calc'd for $C_{23}H_{23}F_2N_4O_4$ $[M+H]^+$ 457.2, found 457.2; $[\alpha]_{D22}$ +71.5° (C 0.2, MeOH); $[\alpha]_{D22}$ +106.8° (C 0.1, $CHCl_3$). Absolute configuration assigned via single crystal X-ray structure determination of the derived intermediate **(+)-SI13**.

And **(-)-SI10** (2.20 g, 45%, peak 2). ^{1}H NMR (400 MHz, DMSO-d_6) δ 7.87 (s, 1H), 7.82 (s, 1H), 7.50 (dd, *J*=5.5, 8.7 Hz, 2H), 7.19 - 7.06 (m, 2H), 6.74 (dd, *J*=2.4, 10.9 Hz, 1H), 6.56 (dd, *J*=2.4, 8.4 Hz, 1H), 5.29 - 4.91 (m, 2H), 4.37 (t, *J*=5.1 Hz, 1H), 3.98 - 3.86 (m, 2H), 3.76 - 3.70 (m, 3H), 3.28 (q, *J*=5.6 Hz, 2H), 1.57 - 1.41 (m, 1H), 1.39 - 1.25 (m, 1H), 1.24 - 1.03 (m, 2H); ^{19}F NMR (376 MHz, DMSO-d_6) δ -102.8 (s, 1F), -113.6 (s, 1F); LRMS (APCI+) m/z calc'd for $C_{23}H_{23}F_2N_4O_4$ $[M+H]^+$ 457.2, found 457.2; $[\alpha]_{D22}$ -89.1° (C 0.2, MeOH); $[\alpha]_{D22}$ -101.5° (C 0.2, $CHCl_3$).

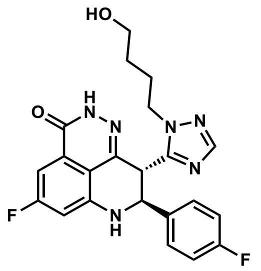

(+)-(8*S*,9*R*)-5-fluoro-8-(4-fluorophenyl)-9-[1-(4-hydroxybutyl)-1*H*-1,2,4-triazol-5-yl]-2,7,8,9-tetrahydro-3*H*-pyrido[4,3,2-*de*]phthalazin-3-one ((+)-SI11). To a room temperature solution of **(+)-SI10** (3.80 g, 8.33 mmol, 1.00 equiv.) in ACN (42 ml, 0.2 M) was added hydrazine monohydrate (1.03 ml, 20.8 mmol, 2.50 equiv.) and the resulting mixture was stirred for 24 h. The reaction mixture was concentrated under reduced pressure (removed ~1/2 volume), diluted with water (60 ml), stirred for 30 min, and filtered. The solids were washed with water (25 ml) and dried to give the title compound (**SI11**) as a white solid (3.04 g, 83%). ^{1}H NMR (400 MHz, DMSO-d_6) δ 12.32 (s, 1H), 7.84 (s, 1H), 7.72 (s, 1H), 7.47 (dd, *J*=5.6, 8.6 Hz, 2H), 7.14 (t, *J*=8.9 Hz, 2H), 7.07 (dd, *J*=2.4, 8.9 Hz, 1H), 6.92 (dd, *J*=2.4, 11.2 Hz, 1H), 5.09 - 4.87 (m, 2H), 4.36 (t, *J*=5.0 Hz, 1H), 3.96 (qd, *J*=7.1, 14.2 Hz, 2H), 3.30 - 3.22 (m, 2H), 1.59 - 1.44 (m, 1H), 1.42 - 1.29 (m, 1H), 1.27 - 1.06 (m, 2H); ^{19}F NMR (376 MHz, DMSO-d_6) δ =-104.2 (s, 1F), -113.7 (s, 1F); ^{13}C NMR (101 MHz, DMSO-d_6) δ 166.1, 163.6, 163.1, 160.7, 158.8 (d, *J*=3.7 Hz,

1C), 151.5, 150.4, 148.4 (d, *J*=12.5 Hz, 1C), 141.3, 135.4 (d, *J*=2.9 Hz, 1C), 130.1 (d, *J*=8.1 Hz, 1C), 129.8 (d, *J*=11.0 Hz, 1C), 115.0 (d, *J*=21.3 Hz, 1C), 111.2, 102.7 (d, *J*=26.4 Hz, 1C), 98.4 (d, *J*=24.2 Hz, 1C), 60.1, 58.9, 47.1, 42.8, 28.9, 26.3; HRMS (APCI+) m/z calc'd for $C_{22}H_{21}F_2N_6O_2$ $[M+H]^+$ 439.1689, found 439.1689; $[\alpha]_{D22}$ = +71.4° (C 0.2, MeOH); $[\alpha]_{D22}$ +88.1° (C 0.2, $CHCl_3$).

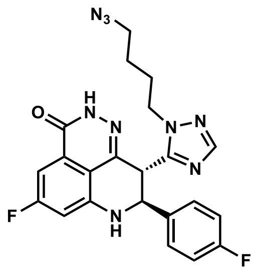

(8*S*,9*R*)-9-[1-(4-azidobutyl)-1*H*-1,2,4-triazol-5-yl]-5-fluoro-8-(4-fluorophenyl)-2,7,8,9-tetrahydro-3*H*-pyrido[4,3,2-*de*]phthalazin-3-one (SI12). To a solution of **SI11** (500 mg, 1.14 mmol, 1.00 equiv) in THF (11.4 ml, 0.1 M) was added DIPEA (397 µL, 2.28 mmol, 2.00 equiv.) followed by MsCl (133 µL, 1.71 mmol, 1.50 equiv.) in a dropwise manner over 1 min. After 2.5 h, the reaction mixture was diluted with EtOAc (120 ml), and the organic layer was washed with water (2 x 20 ml) and brine (15 ml), dried (Na_2SO_4), and evaporated to give the crude mesylate as a white solid that was used without further purification (584 mg, 99%). ^1H NMR (400 MHz, DMSO-d_6) δ 12.33 (s, 1H), 7.87 (s, 1H), 7.73 (s, 1H), 7.48 (dd, *J*=5.6, 8.6 Hz, 2H), 7.16 (t, *J*=8.8 Hz, 2H), 7.07 (dd, *J*=2.4, 8.9 Hz, 1H), 6.93 (dd, *J*=2.4, 11.2 Hz, 1H), 5.08 - 4.94 (m, 2H), 4.10 (t, *J*=6.0 Hz, 2H), 4.05 - 3.99 (m, 2H), 3.13 (s, 3H), 1.67 - 1.53 (m, 1H), 1.51 - 1.32 (m, 3H); ^{19}F NMR (376 MHz, DMSO-d_6) δ -104.2 (s, 1F), -113.6 (s, 1F); LRMS (APCI+) m/z calc'd for $C_{23}H_{23}F_2N_6O_4S$ $[M+H]^+$ 517.1, found 517.2.

To a solution of the crude mesylate (580 mg, 1.12 mmol, 1.00 equiv.) in DMF (5.6 ml, 0.2 M) was added sodium azide (372 mg, 5.61 mmol, 5.00 equiv.). After 2.5 days, the reaction mixture was diluted with EtOAc (150 ml) and water (30 ml) and the layers separated. The aqueous layer was extracted with additional EtOAc (100 ml). The combined organic layers were washed with water (2 x 20 ml) and brine (20 ml), dried (Na_2SO_4), and evaporated to give a crude azide (**SI12**) that was used without further purification (assumed theoretical yield). ^1H NMR (400 MHz, DMSO-d_6) δ 12.31 (s, 1H), 7.86 (s, 1H), 7.71 (s, 1H), 7.48 (dd, *J*=5.5, 8.7 Hz, 2H), 7.20 - 7.11 (m, 2H), 7.07 (dd, *J*=2.4, 8.9 Hz, 1H), 6.93 (dd, *J*=2.4, 11.2 Hz, 1H), 5.10 - 4.90 (m, 2H), 4.12 - 3.89 (m, 2H), 3.21 (t, *J*=6.8 Hz, 2H), 1.61 - 1.47 (m, 1H), 1.46 - 1.36 (m, 1H), 1.35 - 1.19 (m, 2H); ^{19}F NMR (376 MHz, DMSO-d_6) δ -104.2 (s, 1F), -113.7 (s, 1F); LRMS (APCI+) m/z calc'd for $C_{22}H_{20}F_2N_9O$ $[M+H]^+$ 464.2, found 464.3.

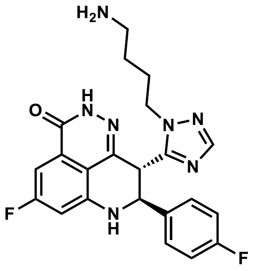

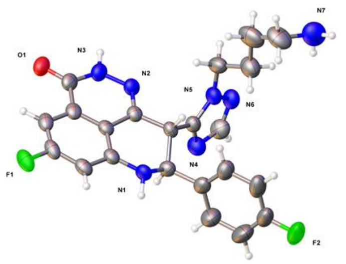

(+)-(8*S*,9*R*)-9-[1-(4-aminobutyl)-1*H*-1,2,4-triazol-5-yl]-5-fluoro-8-(4-fluorophenyl)-2,7,8,9-tetrahydro-3*H*-pyrido[4,3,2-*de*]phthalazin-3-one ((+)-SI13). To a solution of crude azide (**SI12**, ~1.12 mmol) in THF (20 ml) was added water (5 ml) and triphenyl phosphine (363 mg, 1.34 mmol, 1.20 equiv.). The reaction mixture was stirred under nitrogen and heated to 60 °C for 4 h. The reaction mixture was then concentrated, dissolved in MeOH (10 ml) and purified by SFC (ZymorSPHER HADP 21 x 150 mm 5u column at 35 °C eluted with gradient of 12-55% MeOH in CO_2 over 5.25 minutes held at 120 bar, 60 ml/min) to give the title product as a solid (355 mg, 73%). Single crystal X-ray structure analysis of this compound allowed the assignment of absolute configuration. ^{1}H NMR (400 MHz, DMSO-d_6, 80 °C) δ 7.80 (s, 1H), 7.50 (s, 1H), 7.49 - 7.44 (m, 2H), 7.18 - 7.09 (m, 2H), 7.09 - 7.06 (m, 1H), 6.95 (dd, *J*=2.4, 11.2 Hz, 1H), 5.12 - 4.75 (m, 2H), 4.04 - 3.92 (m, 2H), 2.57 (br. s, 1H), 1.60 (d, *J*=4.9 Hz, 1H), 1.52 (br. s, 1H), 1.36 - 1.25 (m, 2H), 1.06 (d, *J*=6.1 Hz, 1H); ^{19}F NMR (376 MHz, DMSO-d_6, 80 °C) δ -104.5 (s, 1F), -113.9 (s, 1F); LRMS (APCI+) m/z calc'd for $C_{22}H_{22}F_2N_7O$ $[M+H]^+$ 438.2, found 438.2; $[\alpha]_{D22}$ +64.3° (C 0.1, MeOH); $[\alpha]_{D22}$ +77.4° (C 0.1, $CHCl_3$).

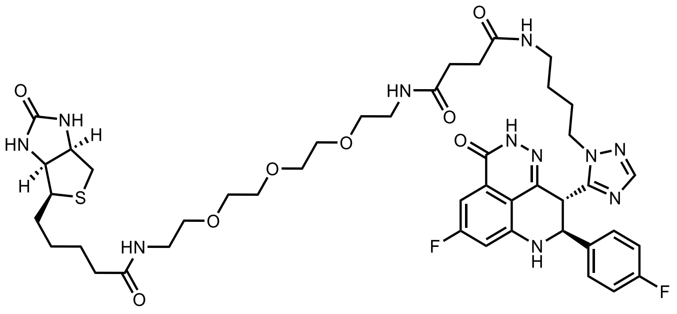

***N*-(4-{5-[(8*S*,9*R*)-5-fluoro-8-(4-fluorophenyl)-3-oxo-2,7,8,9-tetrahydro-3*H*-pyrido[4,3,2-*de*]phthalazin-9-yl]-1*H*-1,2,4-triazol-1-yl}butyl)-*N*'-{13-oxo-17-[(3*aS*,4*S*,6*aR*)-2-oxohexahydro-1*H*-thieno[3,4-*d*]imidazol-4-yl]-3,6,9-trioxa-12-azaheptadec-1-yl}butanediamide (talazoparib-biotin).** To a solution of amine **(+)-SI13** (106 mg, 0.24 mmol, 1.00 equiv.), acid **SI1** (143 mg, 0.28 mmol, 1.20 equiv.), and TEA (101 µl, 0.73 mmol, 3.00 equiv.) in DMF (2.4 ml, 0.1M) was added T3P (50 wt% in EtOAc, 354 µl, 0.61 mmol, 2.50 equiv.) in a dropwise manner over 1 min. After 20 h, the reaction mixture was diluted with *n*-butanol (40 ml), water (10 ml), and saturated aqueous $NaHCO_3$ (10 ml) and agitated. The layers were separated, and the aqueous layer was extracted with additional *n*-butanol (40 ml). The combined organic layers were dried (Na_2SO_4), and evaporated (~2 mmHg, 40 °C) to give a residue, which was purified by SFC (HA-Morpholine column with MeOH and 10 mM NH_3) to give the title product as a white solid (25.7 mg, 11%). ^{1}H NMR (600 MHz, DMSO-d_6) δ 12.30 (br. s, 1H), 7.87 - 7.83 (m, 2H), 7.81 (br. t, *J*=5.6 Hz, 1H), 7.75 (br. t, *J*=5.6 Hz, 1H), 7.71 (s, 1H), 7.47 (dd, *J*=5.6, 8.3 Hz, 2H), 7.14 (br. t, *J*=8.6 Hz, 2H), 7.06 (dd, *J*=2.2, 8.8 Hz, 1H), 6.93

(dd, *J*=2.2, 11.2 Hz, 1H), 6.39 (s, 1H), 6.34 (s, 1H), 5.03 - 4.99 (m, 1H), 4.97 - 4.93 (m, 1H), 4.32 - 4.28 (m, 1H), 4.15 - 4.11 (m, 1H), 3.96 (br. t, *J*=7.2 Hz, 2H), 3.50 (br. d, *J*=2.9 Hz, 8H), 3.38 (q, *J*=6.0 Hz, 4H), 3.17 (quin, *J*=6.1 Hz, 4H), 3.11 - 3.07 (m, 1H), 2.99 - 2.87 (m, 2H), 2.81 (dd, *J*=5.0, 12.4 Hz, 1H), 2.58 (d, *J*=12.5 Hz, 1H), 2.54 (s, 1H), 2.29 - 2.24 (m, 3H), 2.06 (t, *J*=7.4 Hz, 2H), 1.65 - 1.57 (m, 1H), 1.54 - 1.41 (m, 4H), 1.40 - 1.25 (m, 3H), 1.21 - 1.10 (m, 2H); ^{19}F NMR (565 MHz, DMSO-d_6) δ -104.2 (s, 1F), -113.6 (s, 1F); ^{13}C NMR (151 MHz, DMSO-d_6) δ 172.1, 171.4, 171.2, 165.7, 163.4 (d, *J*=211.2 Hz, 1C), 161.1 (d, *J*=244.4 Hz, 1C), 158.8 (d, *J*=3.3 Hz, 1C), 151.6, 150.4, 148.4 (d, *J*=12.2 Hz, 1C), 141.3, 135.4 (d, *J*=2.2 Hz, 1C), 130.1 (br. d, *J* = 7.7 Hz, 1C), 129.8 (d, *J*=11.1 Hz, 1C), 115.1, 115.0, 111.2, 102.9, 102.7, 98.5, 98.4, 69.7, 69.5, 69.1, 69.1, 61.0, 59.2, 58.9, 55.4, 46.8, 42.7, 40.4, 40.0, 38.5, 38.4, 37.8, 35.1, 30.7, 30.7, 28.1, 28.0, 26.9, 25.9, 25.2; HRMS (ESI+) m/z calc'd for $C_{44}H_{57}F_2N_{11}O_8S$ $[M+H]^+$ 938.4153, found 938.4185.

NMR Spectra

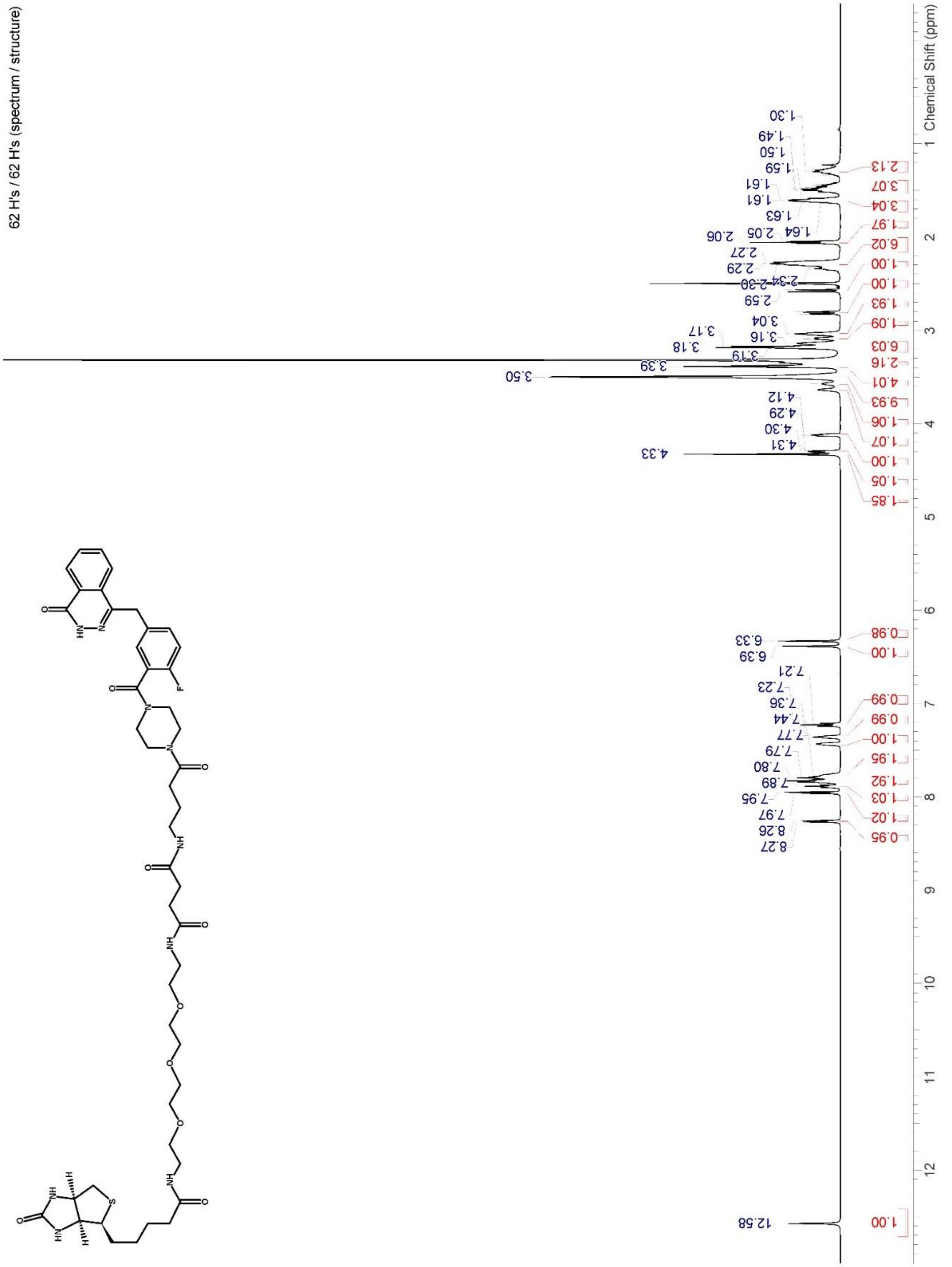

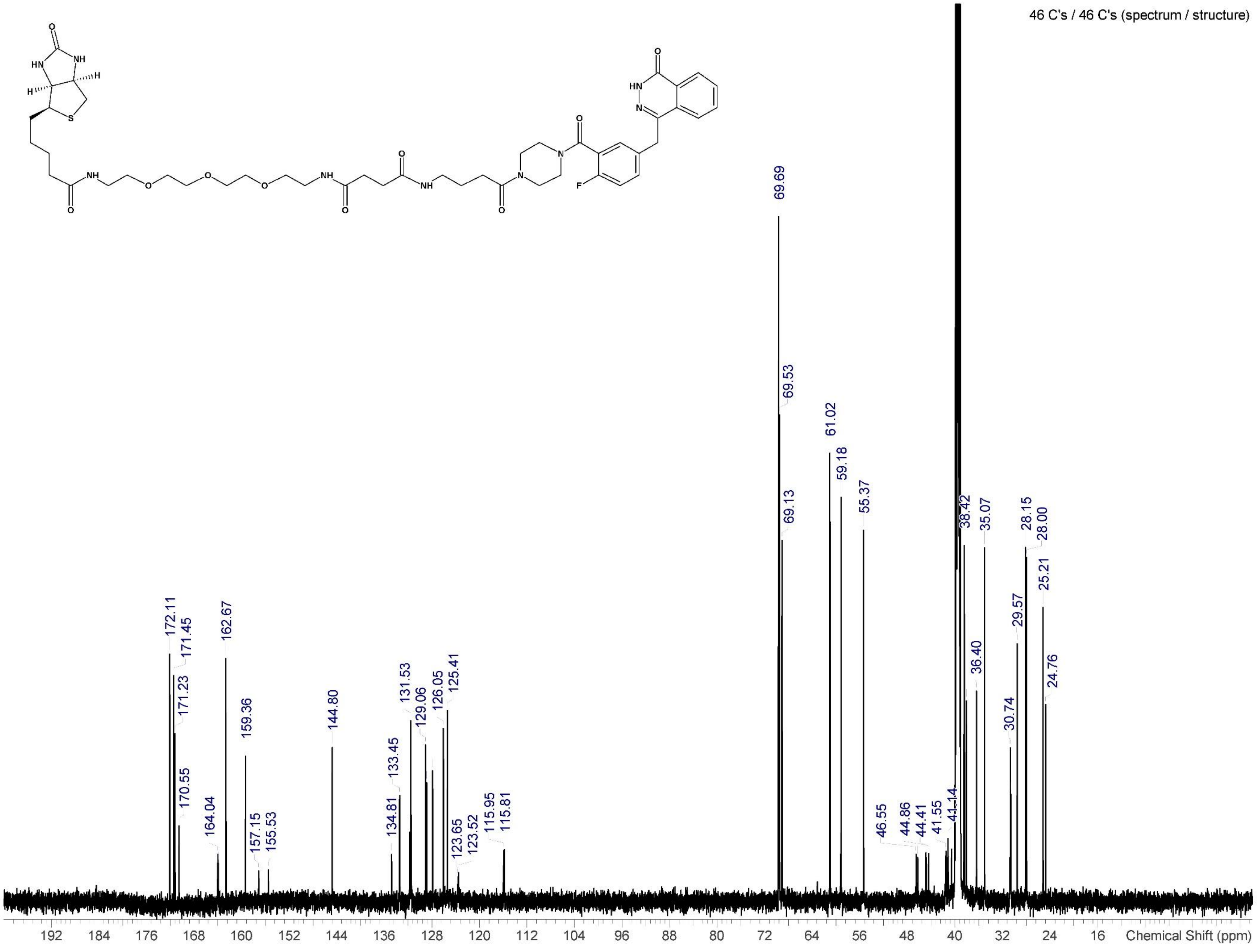

46 C's / 46 C's (spectrum / structure)
172.11
171.45
171.23
170.55
164.04
162.67
159.36
157.15
155.53
144.80
134.81
133.45
131.53
129.06
126.05
125.41
123.65
123.52
115.95
115.81
69.69
69.53
69.13
61.02
59.18
55.37
46.55
44.86
44.41
41.55
41.14
38.42
36.40
35.07
30.74
29.57
28.15
28.00
25.21
24.76
192 184 176 168 160 152 144 136 128 120 112 104 96 88 80 72 64 56 48 40 32 24 16
Chemical Shift (ppm)

57 H's / 57 H's (spectrum / structure)

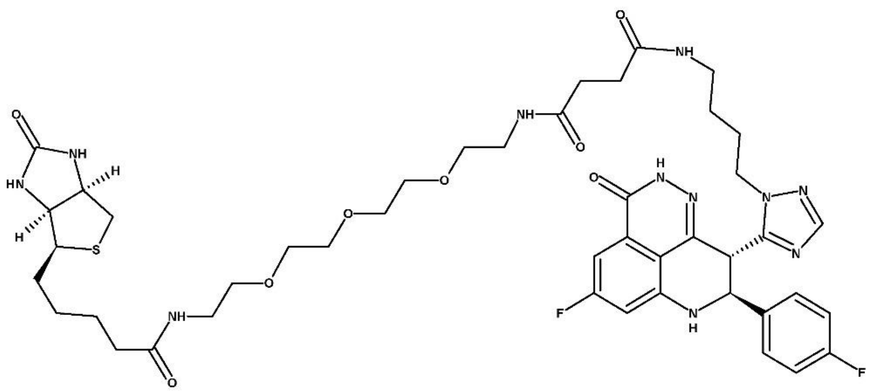

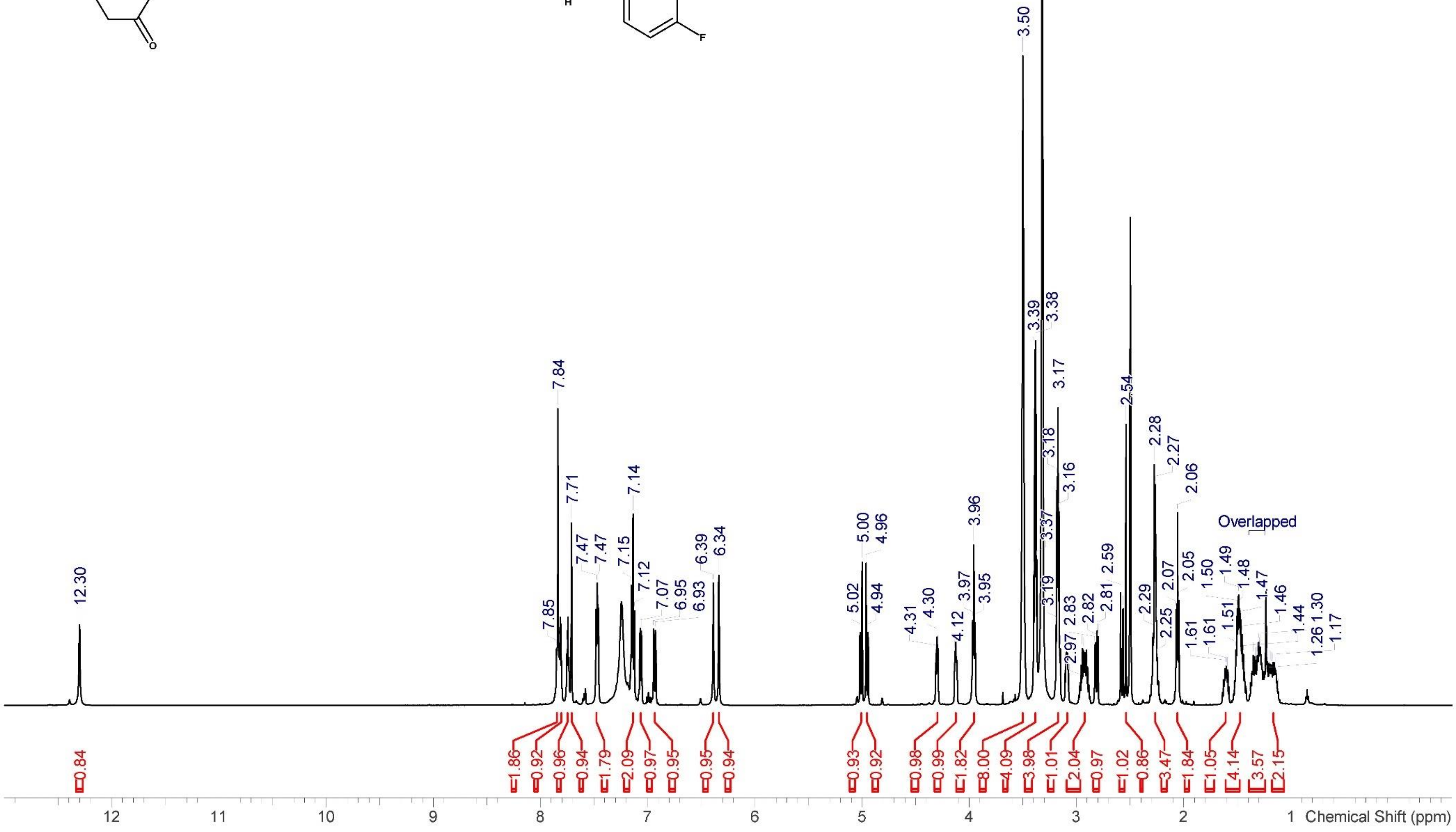

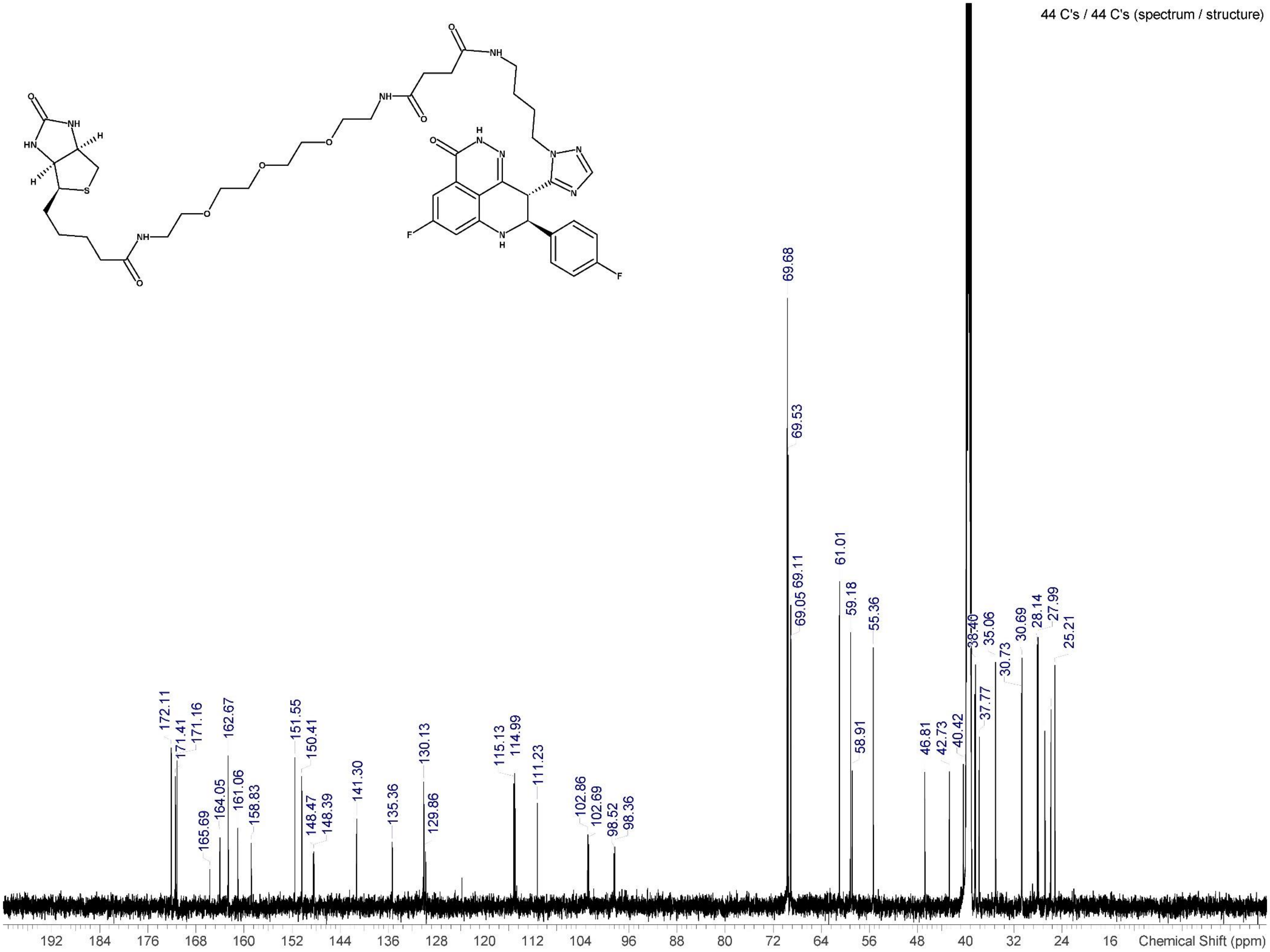
44 C's / 44 C's (spectrum / structure)
172.11
171.41
171.16
165.69
164.05
162.67
161.06
158.83
151.55
150.41
148.47
148.39
141.30
135.36
130.13
129.86
115.13
114.99
111.23
102.86
102.69
98.52
98.36
69.68
69.53
69.05 69.11
61.01
59.18
58.91
55.36
46.81
42.73
40.42
38.40
37.77
35.06
30.73
30.69
28.14
27.99
25.21
Chemical Shift (ppm)
